# Density Functional Theory Investigations on the Mechanism of Formation of Pa(V) Ion in Hydrous Solutions

**DOI:** 10.3390/molecules24061169

**Published:** 2019-03-25

**Authors:** Jun Ma, Chuting Yang, Jun Han, Jie Yu, Sheng Hu, Haizhu Yu, Xinggui Long

**Affiliations:** 1 Institute of Nuclear Physics and Chemistry, China Academy of Engineering Physics, Mianyang 621900, Sichuan, China; majunweixing@126.com (J.M.); yangchuting@caep.cn (C.Y.); junhan966526@126.com (J.H.); husheng@126.com (S.H.); 2 Department of Chemistry and Center for Atomic Engineering of Advanced Materials, Anhui University, Hefei 230601, China; yuhaizhu@ahu.edu.cn

**Keywords:** Pa(V), hydrous solutions, density functional theory (DFT), mechanism

## Abstract

Due to the enormous threat of protactinium to the environment and human health, its disposal and chemistry have long been important topics in nuclear science. [PaO(H_2_O)_6_]^3+^ is proposed as the predominant species in hydrous and acidic solutions, but little is known about its formation mechanism. In this study, density functional theory (DFT) calculations demonstrate a water coordination-proton transfer-water dissociation mechanism for the formation of PaO^3+^ in hydrous solutions. First, Pa(V) ion preferentially forms hydrated complexes with a coordination number of 10. Through hydrogen bonding, water molecules in the second coordination sphere easily capture two protons on the same coordinated H_2_O ligand to form [PaO(H_2_O)_9_]^3+^. Water dissociation then occurs to generate the final [PaO(H_2_O)_6_]^3+^, which is the thermodynamic product of Pa(V) in hydrous solutions.

## 1. Introduction

The traditional uranium-plutonium fuel cycle is faced with many problems, such as the depletion of uranium fuel, the high cost of materials, the tendency to produce fission products with long half-lives, and the post-processing difficulties [1,2]. In recent years, the thorium-uranium fuel cycle has attracted increasing attention because of the abundant thorium resources [3,4,5], the formation of mostly short half-life fission products and relatively easy post-treatment. Under irradiation, the thorium-uranium fuel cycle occurs easily via the nuclear reaction of Th→Pa→U [6], and the main product ^233^U is a frequently used reactor nuclear fuel.

At present, mainly two strategies are used to separate ^233^U. One is to fully cool down after irradiation, and then to separate the generated ^233^U from the irradiated material. The ^233^U extracted by this method contains a little ^232^U, which easily produces ^228^Th nuclides in subsequent treatment. ^228^Th is the main source of irradiation in nuclear fuel reprocessing [6,7]. The other strategy is to firstly separate the intermediate ^233^Pa and store it to cool and naturally decay to ^233^U. The latter method is a better choice because of the higher purity of the separated ^233^U and less long-lived nuclides in use [6,8,9,10]. So far, this method is restricted by the necessity for fast-processing ^233^Pa to reduce the irradiative danger and the possible interference of hazardous ^231^Pa formed in thorium-uranium fuel cycle. To this end, the separation/extraction/stabilization of the highly purified ^233^Pa is fundamentally important. Researches on the chemistry of Pa involved in the extraction/separation processes might provide helpful insights into this topic [11,12,13]. 

In recent years, the coordination structures of protactinium have been reported [14,15,16,17,18,19]. The element Pa mainly shows two possible valence states of Pa(IV) and Pa(V) in hydrous solutions [20]. Pa(IV) is only predominant in highly reductive systems, while Pa(V) is the main species in neutral/acidic hydrous solutions. The +5 charge state and the larger ionic radius of Pa(V) favors the binding of water molecules on the metal center to form a hydrolysis complex [21,22]. Instead of the double M=O bonds frequently reported for other actinide hydrated complexes, formation of only one Pa=O bond reported in the literature [23,24]. However, the reported studies mainly focus on the Pa complexes formed after extraction or separation processes in aqueous solution, whereas how the hydrated structure of Pa(V) ion was formed has been rarely reported [25,26,27]. Considering that the direct binding of O atom to Pa(V) ion is unlikely, we anticipate the Pa=O is formed via a H_2_O coordination-hydrogen transfer mechanism, but the details (e.g., the structure of the precursor, the kinetic profile for the hydrogen transfer etc.) are still unknown. 

In recent studies, the hydrated structure of Pa(V) was mainly deduced from the coordination chemistry of the neighboring actinide elements. For example, the hydrated ions of Pa(V) were suggested to be similar to UO_2_^2+^, with five equatorial water ligands and two axial water molecules [28,29,30]. In accordance with this proposal, the number of water ligands in the PaO^3+^ complex was suggested to be six [23]. By contrast, referring to other actinide species, the number of water molecules in hydrated Pa(V) complexes were also proposed to be higher than seven [23]. For example, the water coordination numbers of Th^4+^, U^4+^, Cm^3+^ and Pu^3+^ were identified to range from 8–12.7, 9–11 [31,32,33,34,35,36,37,38,39,40,41,42,43], nine [44,45] and 10.2 [40,41,42,43] by experimental (such as EXAFS, extended X-ray absorption fine structure) and theoretical (such as density functional theory calculations) methods. The preferential coordination numbers of water molecules with Np^4+^ were suggested to be nine and five [40,41,42,43,46]. Meanwhile, AnO_2_^2+^(VI) and AnO_2_^+^(V) (An=Np, Pu) with five water molecules were relatively stable species in hydrous solution [28,29,30].

According to the above discussions, the coordination number of the hydrated Pa(V) and its accurate structure are still unsettled problems. Specifically, how these structures are finally formed are still unknown. Therefore, we sought probe the formation mechanism of the Pa(V) species in hydrous solutions with density functional theory calculations. This study starts from the possible hydrated structures of Pa(V), and then examined the possible transformations from both kinetic and thermodynamic aspects. Consistent with the recent findings [20,23,24], the hydrated structures of [Pa(H_2_O)_n_]^5+^ (n = 1–12) are unstable, and [Pa=O(H_2_O)_6_]^3+^ are predominant in hydrous solution. The reason mainly relates to the high thermodynamic stability of protactinium complexes, and the high kinetic facility for the hydrogen transfer between the first-coordination-sphere water ligand and the second-coordination-sphere water ligands. 

## 2. Results and Discussion

### 2.1. Structure of hydrated Pa(V) ions

**Figure 1:**
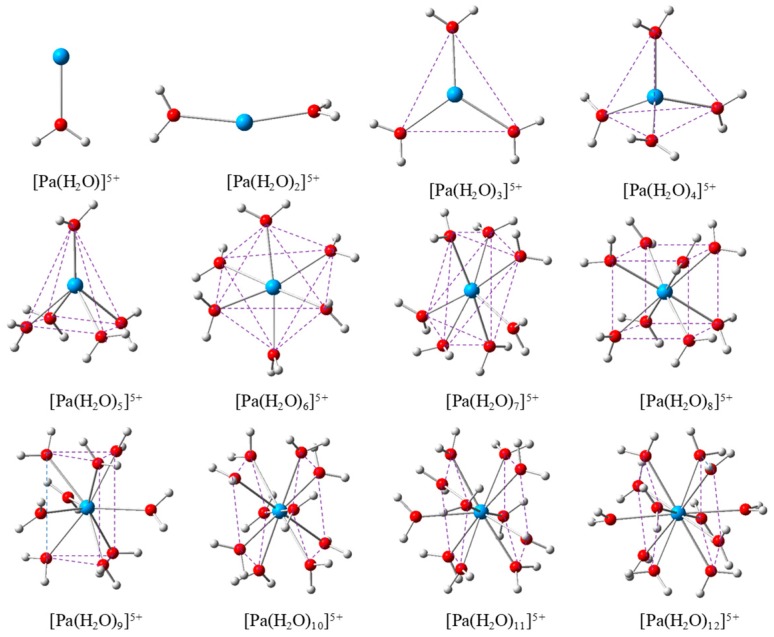
Optimized geometries of [Pa(H_2_O)_n_]^5+^ (n=1-12) calculated with B3LYP/GEN/IEF-PCM level of theory (GEN: SDD for Pa and 6-311G (d, p) for H & O atoms, and IEF-PCM denotes Integral Equation Formalism Polarizable Continuum Model). The blue, red, and white balls denote Pa, O, and H atoms, respectively.

According to the reported hydrated structure of acitinides [23,24,25,26,27,28,29,30,31,32,33,34,35,36,37,38,39,40,41,42,43,44,45,46], 1–14 water molecules could possibly ligate to the Pa(V) centre to form hydrated structures. The DFT calculations indicated the coordination number of Pa(V) is unlikely to exceed 12, because the dissociation of water ligands on the starting geometry of [Pa(H_2_O)_n_]^5+^ (n > 12) inevitably occurs during the geometry optimization (please see the Supplementary Information for more details). Therefore, we suggest that 1–12 are plausible coordination numbers for hydrated Pa(V) structures. Figure 1 shows the optimized structures of these species. 

With only one water ligand, the Pa-O(H_2_O) bond distance of [Pa(H_2_O)]^5+^ is 2.246 Å. [Pa(H_2_O)_2_]^5+^ is a linear-like structure, with the O-Pa-O angle being 164.5°. The [Pa(H_2_O)_3_]^5+^ ion adopts distorted triangular structure, with one O-Pa-O angle being slightly smaller than the other two angles (112.7° vs. 124.3° and 123.0°). [Pa(H_2_O)_4_]^5+^ forms a distorted tetrahedral structure, with three of the four Pa-O bonds slightly longer than the fourth one (2.336, 2.343, 2.344 vs. 2.329 Å). In this regard, Pa(V) does not locate on the centre of the tetrahedron, but closer to one of the facets. [Pa(H_2_O)_5_]^5+^ forms a tetragonal pyramid geometry. The five oxygen atoms locate on the five vertices: four of them on the bottom of the square, while the last oxygen and Pa(V) locate on the normal of this square. [Pa(H_2_O)_6_]^5+^ forms an octahedral-like structure, with the four oxygen atoms on the equatorial plane forming a rectangle, instead of square planar. Pa(V) locates at the intersection of this rectangular diagonal, and the other two water molecules locate on the normal of this rectangular crossing the intersection, one up and one down. To this end, [Pa(H_2_O)_6_]^5+^ shows an approximately central symmetry with Pa(V) as the symmetric centre. The structure of [Pa(H_2_O)_7_]^5+^ is similar to that of [Pa(H_2_O)_6_]^5+^ with six water ligands locating as the vertex of the octahedron, while the last one locates outside one triangular plane as a capping ligand (Figure 1). 

Therefore, it is a formally monocapped octahedral structure. Of note, the geometric structure of [Pa(H_2_O)_7_]^5+^ correlates with the one recently reported by Giandomenico and co-workers [47], but this [Pa(H_2_O)_7_]^5+^ structure is distinct from that of the five coordinated uranyl complexes [28,29,30], which formally adopt a configuration with five equatorial ligand and two axial oxygen atoms. [Pa(H_2_O)_8_]^5+^ forms a cube structure with a central Pa(V) atom. [Pa(H_2_O)_9_]^5+^ forms a tri-capped triangular prism configuration, which is a common geometry for 9-coordinated actinide structures. As shown in Figure 1, the upper three and the lower three water ligands constitute a triangular prism, while the remaining three water ligands locate on the vertical lines of the three side rectangles. This structure is very similar to the structure of [Cm(H_2_O)_9_]^3+^ determined by single-crystal X-ray crystallography [44,45]. [Pa(H_2_O)_10_]^5+^ forms a pentagonal anti-prism, and the ten oxygen atoms are divided into two groups, each five form an pentagon. The [Pa(H_2_O)_10_]^5+^ structure adopts an approximate C_5i_ symmetry, with the Pa(V) acting as the symmetric centre. Compared to [Pa(H_2_O)_10_]^5+^, the additional water ligand on [Pa(H_2_O)_11_]^5+^ acts as a capped ligand on one of the pentagon, and thus the Pa-O bond therein is significantly larger than all the other ones (2.433 vs. 2.480 Å). Due to the incorporation the 11th water ligand, the Pa(V) does not act as the symmetric centre of [Pa(H_2_O)_11_]^5+^. Meanwhile, the 11th water ligand locates on the vertical axis of [Pa(H_2_O)_11_]^5+^, and thus the C_5_ symmetry was maintained. [Pa(H_2_O)_12_]^5+^ adopts an icosahedron-like configuration. Alternatively, the optimized structure of [Pa(H_2_O)_12_]^5+^ could also be viewed as a bicapped pentagonal antiprism, with each pentagon further capped by one water ligand.

### 2.2. Analysis on Pa-O Bond Distance

**Figure 2:**
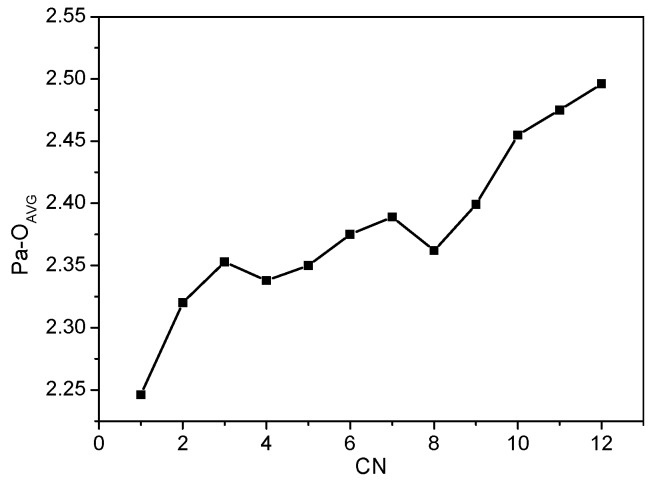
Relationship between Pa-O_AVG_ bond length and the number of water ligands in [Pa(H_2_O)_n_]^5+^ (n = 1–12). The bond distances were read from the B3LYP/GEN/IEF-PCM method optimized geometries (for details see Figure 1 caption).

**Figure 3:**
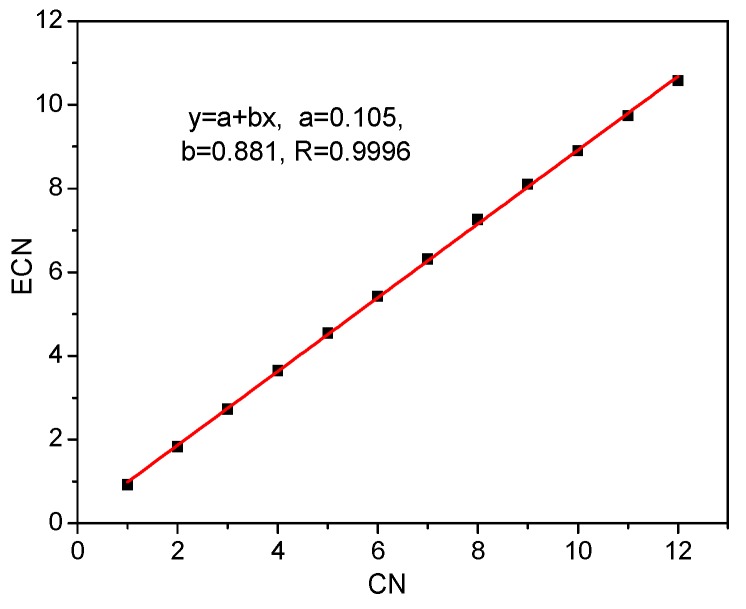
The relationship between effective coordination number and the number of water ligands.

Numbering can be added as follows: the Pa-O distances in [Pa(H_2_O)_n_]^5+^ (n = 1–12) are listed in Table 1. For clarity, the average Pa-O distances in these complexes are designated as Pa-O_AVG_. The trend of Pa-O_AVG_ is shown in Figure 2. According to the calculation results, the Pa-O_AVG_ distance is commonly lengthened with the increase in the water molecule coordination number (Figure 2). However, the Pa-O_AVG_ distances of [Pa(H_2_O)_n_]^5+^ is not always larger than those of [Pa(H_2_O)_n-1_]^5+^. For example, the Pa-O_AVG_ of [Pa(H_2_O)_4_]^5+^ is relatively shorter than that of [Pa(H_2_O)_3_]^5+^. Similarly, Pa-O_AVG_ of [Pa(H_2_O)_8_]^5+^ is relatively shorter than that of [Pa(H_2_O)_7_]^5+^. The reason for such observations mainly relates to the reduced electrostatic repulsions between the lone pairs of different oxygen atoms (on different water ligands). Therefore, the Pa-O_AVG_ distance regularly increases within three groups of [Pa(H_2_O)_n_]^5+^, i.e., n = 1–3; 4–7; 8–12. Specifically, the difference in the Pa-O_AVG_ distance of [Pa(H_2_O)_n_]^5+^and [Pa(H_2_O)_n-1_]^5+^ in these three groups ranges from 0.017 to 0.081 Å. The tiny change indicates that the incorporation of an additional water molecule does not significantly perturb the coordination of existing water molecules. This proposal is further evidenced by the effective coordination number (ECN) analysis (Table 1 and Figure 3). 

As shown in Figure 3, ECN of [Pa(H_2_O)_n_]^5+^ shows an excellent linear correlation with the number of water ligands, with a linear correlation coefficient of 0.9996 and root-mean standard deviation of 0.038. The slope of 0.881 indicates that the efficient coordination efficiency for the Pa-O bond in all these hydrous structures is comparable, and close to 0.9. The efficiency shows little dependency on the coordination number, which is mainly caused by the large atomic radius and high diffusion of the 5f orbitals of the Pa(V) ion.

### 2.3. Thermodynamic analysis

**Figure 4:**
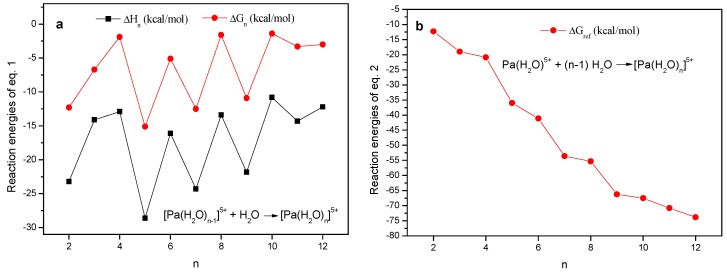
Correlation between thermodynamic data and number of water ligands on [Pa(H_2_O)_n_]^5+^ for Equations (1) (**a**) and (2) (**b**). The enthalpies and Gibbs free energies are calculated with the B3LYP-D3(BJ)/GEN/IEF-PCM//B3LYP/GEN /IEF-PCM method (for details see Table 2 caption).

The reaction energies (including ΔH_n_, ΔG_n_, and ΔS_n_) of Equation (1) was used to evaluate the thermal stability of different hydrated structures of Pa(V). The related results are given in Table 2. For clarity, the trends of the reaction energy of these reactions were drawn in Figure 4a:

[Pa(H_2_O)_n-1_]^5+^+ H_2_O→[Pa(H_2_O)_n_]^5+^ (1)

It can be seen from Table 2 and Figure 4a, ΔH_n_ for all these entries are negative, indicating the exothermic character of all these reactions. Meanwhile, ΔG_n_ shows almost the same trends with ΔH_n_, indicating that the TΔS are comparable in these systems (Table 2). The negative TΔS demonstrates that the water ligation reactions are all disfavoured by the entropic effect, predominantly due to the reduced freedom degree. However, ΔH dominates over TΔS, and thus the reaction Gibbs free energies for all these elementary reactions are negative. Accordingly, the stability of the hydrated ions increased gradually with the increasement of the number of water molecules, and the possibility of the product hydrated structures gradually increases. 

Interestingly, both ΔH_n_ and ΔG_n_ for n ≧ 3 show clear zig-zag profiles. The results indicate that the binding of an additional water on the even water ligated Pa(V) structure is favoured over the water addition processes on odd water ligated structures. We anticipated that the higher symmetry of the hydrated structures with even number of water ligands (compared to those with odd water ligands) results in stronger intramolecular electrostatic repulsion between coordinated ligands and weaker intermolecular Van der Waals interactions, and thus the binding of an extra-water ligand reduces the geometric symmetry and enhanced the Van der Waals interactions.

Using [Pa(H_2_O)]^5+^ as an energy reference, the continuous water coordination results in the formation of [Pa(H_2_O)_n_]^5+^ (n = 2–12, Equation (2)). The Gibbs free energy changes (ΔG_n_^ref^) and the enthalpy changes (ΔH_n_^ref^) for the reactions (2) are given in Table 2, and ΔG_n_^ref^ are used to determine the overall thermodynamic facility of the water binding reactions, and the trends are shown in Figure 4b for clarity reasons. From Figure 4b, the binding of water ligand on Pa(V) ion is continuously favoured. The results seem to suggest that [Pa(H_2_O)_12_]^5+^ is thermodynamically more favoured than the other hydrated structures. However, when extra water molecules are incorporated in the second coordination sphere (to simulate the actual solution-phase environment), the hydrogen-bonding induced by the extra-water molecule easily perturbs the structure of [Pa(H_2_O)_12_]^5+^ and [Pa(H_2_O)_11_]^5+^ to form hydrated Pa(V) structures with only nine or 10 water ligands. The results correlate with the recent studies by Siboulet et al. on the similar uranyl system [48], which indicates that the incorporation of water molecules on the second coordination sphere remarkably affect the vibrational frequencies and U-O bond distances. According to these phenomena, we propose that [Pa(H_2_O)_11_]^5+^ and [Pa(H_2_O)_12_]^5+^ are inaccessible structures in hydrous solutions, and the largest number of water ligands tolerated on hydrated Pa(V) ion should be 10. Because the energy of [Pa(H_2_O)_10_]^5+^ is relatively lower than those of all other hydrated Pa(V) structures (Table 2 and Figure 4b), the transformation from [Pa(H_2_O)_n_]^5+^ to Pa=O species were examined starting from [Pa(H_2_O)_10_]^5+^:

[Pa(H_2_O)]^5+^+(n-1) H_2_O→[Pa(H_2_O)_n_]^5+^ (2)

Equilibrium constants K, K’ for reactions (1) and (2) were listed in Table 2. Formula −ΔG = −RTlnK was used for calculating equilibrium constants, in which R equals to 8.314 J/mol.K, and T equals 298.15K.

### 2.4. Formation of [Pa=O(H_2_O)_x_]^3+^ from [Pa(H_2_O)_n_]^5+^

**Figure 5:**
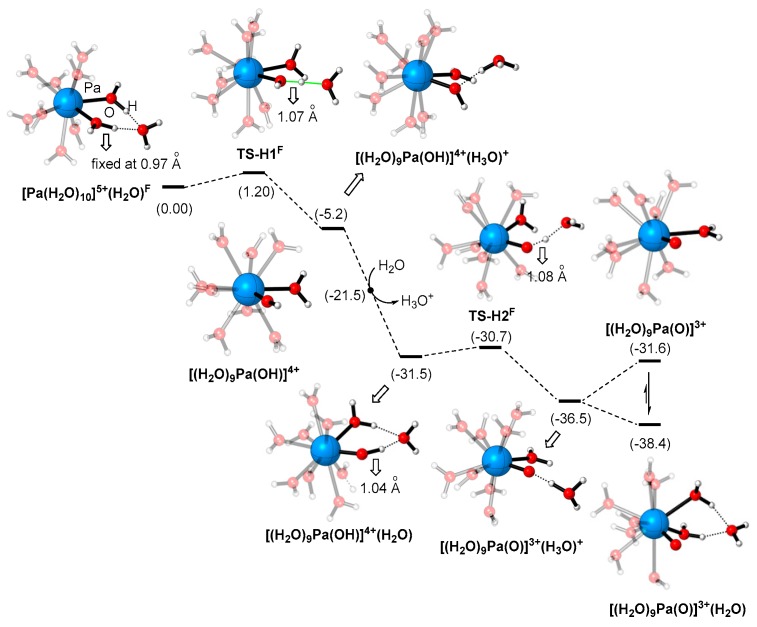
The Gibbs free energy profile for formation of [Pa(H_2_O)_10_]^5+^(H_2_O) to [PaO(H_2_O)_9_]^3+^(H_2_O) calculated with B3LYP-D3(BJ)/GEN/IEF-PCM//B3LYP/GEN /IEF-PCM method (for details see Table 2 caption). Note: the energy changes for the transformations of [Pa(H_2_O)_10_]^5+^(H_2_O)^F^→TS-H1^F^ and [(H_2_O)_9_Pa(OH)]^4+^(H_2_O)→TS-H2^F^ are the total electronic energy changes, because the thermodynamic corrections of the transition state structures obtained by partial optimization are unavailable.

From [Pa(H_2_O)_10_]^5+^, the incorporation of an extra water molecule in the second coordination sphere results in a spontaneous hydrogen transfer during the geometry optimization. The phenomenon originates from both the relatively higher thermodynamic stability of the formed [Pa(OH)(H_2_O)_9_]^4+^(H_3_O)^+^, and the kinetic easiness to overcome the energy barrier of the hydrogen transfer transition state. This assumption is verified by the partial geometry optimization calculations (by fixing the O-H bond at different distances, i.e., 0.97 Å in the free [Pa(H_2_O)_10_]^5+^, 1.07 Å, 1.17 Å, 1.27 Å, 1.37 Å, 1.47 Å, 1.57 Å and 1.67 Å). For clarity reasons, the relative energy of [Pa(H_2_O)_10_]^5+^(H_2_O) is set as the reference state in this section. According to the calculation results (please see Figure S3 for the details): starting from [Pa(H_2_O)_10_]^5+^(H_2_O), the partial optimization energy profile undergoes a tiny barrier (1.2 kcal/mol) at 1.07 Å, after which the energy continuously decreased until the formation of the intermediate [Pa(OH) (H_2_O)_9_]^4+^(H_3_O)^+^ (Figure 5). The relative energy of [Pa(OH) (H_2_O)_9_]^4+^(H_3_O)^+^ is 5.2 kcal/mol lower than that of the starting point, i.e., [Pa(H_2_O)_10_]^5+^(H_2_O). From [Pa(H_2_O)_9_(OH)]^4+^(H_3_O)^+^, the protonated water on the second coordination sphere could be easily exchanged by another water molecule. This process is suggested to occur via a dissociative mechanism, in which the H_3_O^+^ on the second coordination sphere first dissociates to form the intermediate [Pa(OH)(H_2_O)_9_]^4+^ with an energy decrease of 16.3 kcal/mol. After that, the water binding occurs easily to stabilize the protactinium complex via hydrogen bonding, with the formation of [Pa(OH)(H_2_O)_9_]^4+^(H_2_O) as a result. Then, the hydrogen transfer from -OH on Pa to the water on the second coordination sphere occurs easily. The partial geometry optimization (Figure S4) indicates a very low energy barrier of 0.8 kcal/mol when the O-H bond distance reaches about 1.07 Å. [PaO(H_2_O)_9_]^3+^(H_3_O)^+^ was then formed as the product of the second hydrogen transfer process, and its relative energy is 36.5 kcal/mol lower than that of the initial hydrated structure [Pa(H_2_O)_10_]^5+^(H_2_O). Similar to the aforementioned observations, the substitution of the H_3_O^+^ in the second coordination sphere of [PaO(H_2_O)_9_]^3+^(H_3_O)^+^ with H_2_O is exergonic (Figure 5). By contrast, the direct dissociation of the (H_3_O)^+^ is slightly endergonic by 4.9 kcal/mol. Therefore, PaO(H_2_O)_9_]^3+^(H_2_O) functions as the final product of the hydrogen transfer pathway starting from [Pa(H_2_O)_10_]^5+^ (Figure 5).

In Figure 5, the kinetic profile for the transformation of [Pa(H_2_O)_10_]^5+^ to [PaO(H_2_O)_9_]^3+^ was examined, and was found to be highly favoured both kinetically and thermodynamically. Because the relative energy of the other hydrated Pa(V) structures, i.e., [Pa(H_2_O)_n_]^5+^ (n = 1–9) is significantly higher than that of [Pa(H_2_O)_10_]^5+^ by over 1.2 kcal/mol (the energy demand for the overall transformations in Figure 5), the direct participation of all other hydrated structures could be excluded.

**Figure 6:**
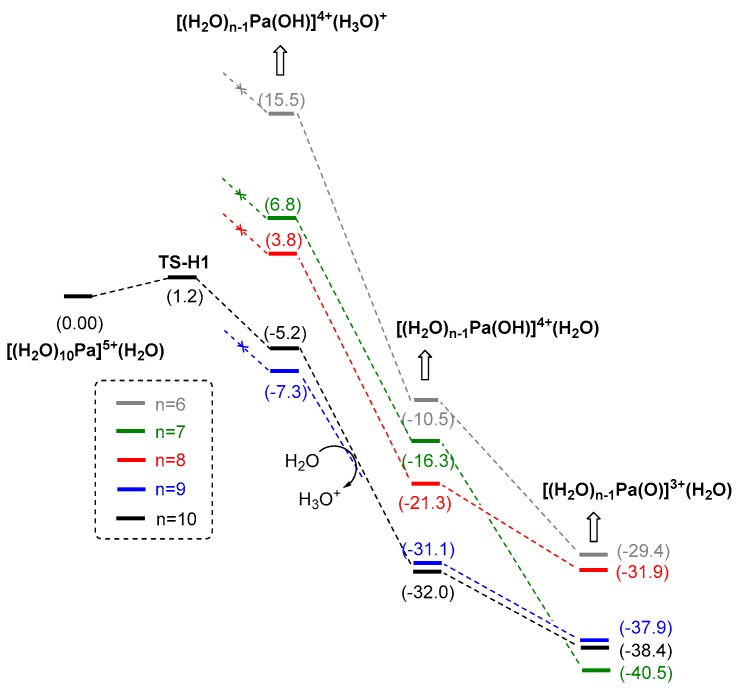
Comparison of Gibbs free energy profiles starting from [Pa(H_2_O)_n_]^5+^(H_2_O) (n=6-10) calculated with B3LYP-D3(BJ)/GEN/IEF-PCM//B3LYP/GEN /IEF-PCM method (for details see Table 2 caption).

Nevertheless, due to fast coordination equilibrium in the hydrous solutions (water dissociation, exchange, and re-coordination) [30,46,48,49,50], the formation of other intermediates with fewer water ligands (compared to the related ones in Figure 5) cannot not be precluded. Therefore, the key intermediates, including [Pa(OH)(H_2_O)_n-1_]^4+^(H_3_O)^+^, [PaO(H_2_O)_n-1_]^3+^(H_3_O)^+^, and [PaO(H_2_O)_n-1_]^3+^(H_2_O) with n = 6–9 were also examined. The detailed results, associated with the results starting from [Pa(H_2_O)_10_]^5+^(H_2_O) are provided in Figure 6 for comparison. From Figure 6, the relative energy for the species of n = 9 are always comparable to that of n = 10, indicating the co-existence of these species. According to these results, despite the direct proton transfer on [Pa(H_2_O)_9_]^5+^ is unlikely, the intermediates involved in Figure 5 might undergo fast water dissociation and re-coordination, and thus both the black (n = 10) and the blue (n = 9) pathways are responsible for the formation of the Pa=O products. By contrast, when n = 6–8, [Pa(OH)(H_2_O)_n-1_]^4+^(H_3_O)^+^ and [PaO(H_2_O)_n-1_]^3+^(H_3_O)^+^ are all unlikely to be formed, due to the remarkably higher energy of these species compared the related ones when n = 9/10. However, for the final Pa=O product, it is interesting to note that the most stable structure is [PaO(H_2_O)_6_]^3+^(H_2_O) (when n = 7, Figure 6), whose energy is over 2 kcal/mol lower than all other [PaO(H_2_O)_n-1_]^3+^(H_2_O) (n = 6, 8–10) species. As [PaO(H_2_O)_6_]^3+^(H_2_O) could be easily formed via the water dissociation from [PaO(H_2_O)_8/9_]^3+^(H_2_O), we suggest that the predominant hydrated structure of Pa(V) is [PaO(H_2_O)_6_]^3+^(H_2_O). A small amount of [PaO(H_2_O)_8/9_]^3+^(H_2_O) might also co-exist with [PaO(H_2_O)_6_]^3+^(H_2_O), due to the small energy gap between these species. In the recent study reported by Toraishi and co-workers [23], [PaO(H_2_O)_6_]^3+^ was also suggested to be the most stable structure of PaO^3+^ hydrated ions.

Note that in our calculations, we also examined the possibility for the formation of the [(H_2_O)_n-1_Pa(OH)_2_]^3+^(H_3_O)^+^ structures via the H-transfer from the first-coordination-sphere water ligand of [(H_2_O)_n_Pa(OH)]^4+^(H_2_O) to the second-coordination-sphere H_2_O. The partial optimization starting from [(H_2_O)_9_Pa(OH)]^4+^(H_2_O) indicated a clear energy barrier of about 2 kcal/mol, which is relatively disfavoured compared to the <1 kcal/mol energy barrier of the H-transfer from the hydroxide group in Figure 5 (Please see Figure S6 for the details).

**Figure 7:**
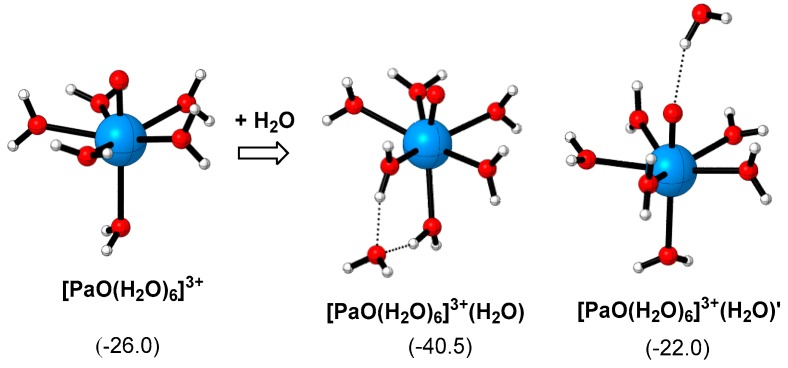
Optimized geometries and relative Gibbs free energies of [PaO(H_2_O)_6_]^3+^, [PaO(H_2_O)_6_]^3+^(H_2_O) and [PaO(H_2_O)_6_]^3+^(H_2_O)’, the blue, red, and white balls denote Pa, O, and H atoms, respectively. The energies are gained with the B3LYP-D3(BJ)/GEN/IEF-PCM//B3LYP/GEN /IEF-PCM method (for details see Table 2 caption).

Finally, efforts were made to probe the geometric and electronic structure of [PaO(H_2_O)_6_]^3+^ and [PaO(H_2_O)_6_]^3+^(H_2_O). [PaO(H_2_O)_6_]^3+^ formally adopts a pentagonal bipyramidal geometry (Figure 7) [23]. The Pa=O distance is significantly shorter than the equatorial Pa-O bond distances (~1.80 vs. ~2.45 Å in average), while the axial Pa-O distance trans- to the Pa=O is relatively longer than the equatorial Pa-O distances by about 0.2 Å (Figure S5). Meanwhile, the NBO charge on the axial water ligand also deviates from those on the equatorial ligands (−0.850 vs. −0.876 in average, Table S1), indicating the weaker electron donating ability of the axial water and the weaker Pa-O bond therein (supported by Wiberg bond index (WBI) analysis, Table S1). To this end, we suggest that the axial water is the most active ligand on the hydrated structure of Pa(V), and is thus labile to be replaced during the extraction processes [51,52,53]. With the incorporation of an extra water molecule in the second coordination sphere, [PaO(H_2_O)_6_]^3+^(H_2_O) could be easily formed to stabilize the protactinium complex. This process is exergonic by 14.5 kcal/mol, which is caused by the hydrogen bonding between the second-coordination-sphere water and the first-coordination-sphere water ligands. However, the key Pa=O and Pa-O distances were hardly perturbed by the second-coordination-sphere water molecule, because the related Pa-O/Pa=O bond distances are alike in these two molecules (~1.80 Å for Pa=O, ~2.45 Å for equatorial Pa-O, and ~2.60 Å for axial Pa-O distance, please see Figure S5 for details). Therefore, the second-coordination-sphere water makes little influence on the Pa-O/Pa=O bond order, strength and their chemistry.

Despite the fact the second-coordination-sphere water molecule does not affect the relative Pa-O/Pa=O binding strength in the first coordination sphere, some interesting observations regarding the orientation of the second-coordination-sphere water molecule were noted. As shown in Figure 7, the relative energy of another typical isomer, i.e., [PaO(H_2_O)_6_]^3+^(H_2_O)’, is significantly higher than that of [PaO(H_2_O)_6_]^3+^(H_2_O). The results indicate that the type and position of the second-coordination-sphere water molecules are pivotal to the thermodynamic stability of the protactinium complexes. The main reason for the instability of [PaO(H_2_O)_6_]^3+^(H_2_O)’ relates to the weakened Pa=O bond strength (evidenced by the relatively longer Pa=O distance, Figure S5). The results correlate with the recent studies on the complex solvent effect of the second and even third coordination sphere in actinide systems [45,54,55]. Motivated by these observations, we anticipated that in actual hydrous solutions, the solvent molecules could constitute complex hydrogen bonding interactions with the central protactinium complex. More efforts are currently underway in our group to elucidate the detailed solvent effect.

## 3. Computation Methods 

As the B3LYP method has been frequently used to treat actinide systems [23], the geometry optimization for all structures in this study were carried out using B3LYP functional in Gaussian 09 program [56]. The full electron 6-311G (d, p) basis set was used to treat the O and H elements, while the small-core pseudo potential of SDD basis set was used to treat the actinides element Pa, assuming the 60 electrons on inner layers of Pa as the core electrons [57,58]. As the relative energy of the +5, +4 or +3 charged Pa(V) species and the energy demand of the related charge-separation processes (please see supporting information for details) are highly sensitive to the solvent effect, all geometry optimizations were conducted using the Integral Equation Formalism Polarizable Continuum Model (IEF-PCM) solvation model [59,60,61] with water as the solvent. The same functional and basis set were used for the vibrational analysis, to verify the gained structures are local minima and to gain the thermal correction data. The temperature used for all calculations are 298.15 K. Based on the optimized structures, the Becke and Johnson (BJ) corrections as described by Grimme [62] were used to incorporate the dispersion interactions, and better describe the energy profiles. Note that the incorporation of the Douglas-Kroll-Hess 2^nd^ order scalar relativistic (DKH for short) in the single point energy calculations does not change our conclusions (Table S2). The NBO charge [63,64,65,66,67,68,69], effective coordination number [70], Wiberg Bond Index (WBI) bond order [71] and Kohn-Sham orbital analysis were also performed with the same method. To test the influence of the relativistic, we used the Douglas-Kroll-Hess 2^nd^ order scalar relativistic (with the abbreviation of DKH) to calculate the energy demands for formation of the possible hydrated ions ([PaO(H_2_O)_n_]^3+^(H_2_O), n = 5–9) and [Pa(H_2_O)_9_]^5+^. The additional calculations showed consistency with those in Figure 6, and the details are given in supporting formation (Table S2). 

The different spin states (singlet, triplet, quintet, septet) of selected Pa(V) species ([Pa(H_2_O)_5_]^5+^ and [Pa(H_2_O)_7_]^5+^) are calculated [23]. The relative energy of the singlet state was found to be the lowest in both cases, and thus all other Pa(V) structures are calculated with singlet state. The effective coordination number (ECN) are calculated according to Equation (3) [70], in which dM_jsp_ corresponds to the Pa-O bond distance Tn Table 1, k equals to 2.0 a.u, and d^0^ equals to 3.5 Å:

(3) $$\mathrm{ECN}=\sum_{jsp=1}^{N_{sp}}\frac{1}{1+e^{k\left(dM_{jsp}-d^{0}\right)}}$$

## 4. Conclusions

The chemistry and extraction of Pa(V) has been a long attractive but challenging problem in recent decades. In this study, density functional theory (DFT) calculations were conducted to elucidate the initial state of the normal Pa(V) in aqueous solutions, and their kinetic/thermodynamic profiles to the formation of protactinium (Pa=O) complexes. According to the calculation results, the following conclusions are obtained:

(1) The maximum allowed coordination number of water ligands on the Pa(V) centre is 10.
(2) [Pa(H_2_O)_10_]^5+^ is the initial species in aqueous solution, but is labile to the proton transfer with the water ligands on the second coordination sphere, to form the stable Pa=O complexes with higher thermodynamic stability. The transformations are kinetically highly feasible (with energy demand < 1.5 kcal/mol).
(3) The most stable Pa=O structure in aqueous solution is [PaO(H_2_O)_6_]^3+^. This structure is formed by the proton transfer-water dissociation mechanism starting from [Pa(H_2_O)_10_]^5+^.
(4) [PaO(H_2_O)_6_]^3+^ adopts a pentagonal bipyramidal geometry. The relatively weaker Pa-O bond trans- to the Pa=O bond (compared to the equatorial Pa-O bonds) results in an easier replacement in the ligand exchange and extraction reactions.

## Figures and Tables

**Table 1:** The Pa-O, Pa-O_AVG_ distances (in Å), effective coordination numbers (ECN) and natural bond orbital (NBO) charge distribution of [Pa(H_2_O)_n_]^5+^ (n = 1–12). All these data were gained with the B3LYP/GEN/IEF-PCM method (for details see Figure 1 caption).

		**[Pa(H_2_O)]^5+^**	**[Pa(H_2_O)_2_]^5+^**	**[Pa(H_2_O)_3_]^5+^**	**[Pa(H_2_O)_4_]^5+^**	**[Pa(H_2_O)_5_]^5+^**	**[Pa(H_2_O)_6_]^5+^**
Bond Distances	Pa-O	2.246	2.320	2.317–2.420	2.329–2.344	2.301–2.391	2.344–2.401
	Pa-O_AVG_	2.246	2.320	2.353	2.338	2.350	2.375
Effective coordination number (ECN)		0.93	1.83	2.73	3.64	4.54	5.43
NBO Charges on	Pa	4.687	4.462	4.239	3.944	3.702	3.365
	O	−0.927	−0.928	−0.910 to −0.928	−0.908 to −0.910	−0.881 to −0.915	−0.872 to −0.893
	O_AVG_	−0.927	−0.928	−0.922	−0.909	−0.906	−0.882
		**[Pa(H_2_O)_7_]^5+^**	**[Pa(H_2_O)_8_]^5+^**	**[Pa(H_2_O)_9_]^5+^**	**[Pa(H_2_O)_10_]^5+^**	**[Pa(H_2_O)_11_]^5+^**	**[Pa(H_2_O)_12_]^5+^**
Bond distances	Pa-O	2.342–2.431	2.347–2.377	2.345–2.450	2.423–2.506	2.433–2.544	2.480–2.517
	Pa-O_AVG_	2.389	2.362	2.399	2.455	2.475	2.496
Effective coordination number (ECN)		6.32	7.26	8.10	8.90	9.74	10.59
NBO Charges on	Pa	3.004	2.288	1.933	1.815	1.371	0.931
	O	−0.851 to −0.870	−0.818 to −0.822	−0.802 to −0.813	−0.802 to −0.808	−0.783 to −0.790	−0.769 to −0.773
	O_AVG_	−0.861	−0.820	−0.807	−0.804	−0.787	−0.771

**Table 2:** The reaction enthalpies, Gibbs free energies, entropies and equilibrium constants for reaction (1) and (2) calculated with B3LYP-D3(BJ)/GEN/IEF-PCM//B3LYP/GEN /IEF-PCM method (for details see Figure 1 caption, BJ = Becke and Johnson correction).

n	Reaction (1)				Reaction (2)		
	ΔH_n_ (kcal/mol)	ΔG_n_ (kcal/mol)	TΔS_n_ (kcal/mol)	K	ΔH_n_^ref^ (kcal/mol)	ΔG_n_^ref^ (kcal/mol)	K’
**2**	−23.2	−12.3	−10.9	1.1 × 10^9^	−23.2	−12.3	1.1 × 10^9^
**3**	−14.1	−6.7	−7.3	8.7 × 10^4^	−37.3	−19.0	8.7 × 10^13^
**4**	−12.9	−1.9	−11.0	2.5 × 10^1^	−50.1	−20.9	2.1 × 10^15^
**5**	−28.6	−15.1	−13.5	1.2 × 10^11^	−78.7	−36.0	2.6 × 10^26^
**6**	−16.1	−5.1	−11.0	5.5 × 10^3^	−94.8	−41.1	1.4 × 10^30^
**7**	−24.3	−12.5	−11.8	1.5 × 10^9^	−119.1	−53.6	2.1 × 10^39^
**8**	−13.4	−1.6	−11.8	1.5 × 10^1^	−132.5	−55.3	3.7 × 10^40^
**9**	−21.8	−10.9	−10.9	9.9 × 10^7^	−154.3	−66.2	3.7 × 10^48^
**10**	−10.8	−1.3	−9.4	9.0 × 10^0^	−165.0	−67.5	3.3 × 10^49^
**11**	−14.3	−3.3	−11.0	2.6 × 10^2^	−179.4	−70.8	8.6 × 10^51^
**12**	−12.2	−3.0	−9.2	1.6 × 10^2^	−191.6	−73.8	1.4 × 10^54^

## Supplementary Materials

The following are available online, Figure S1: Optimized geometries of [Pa(H_2_O)_n_]^5+^ (n = 13,14); Figure S2: Pa-O Wiberg bond index (WBI) of [Pa(H_2_O)_9_]^5+^; Figure S3: Electronic energy profile of the hydrogen transfer starting from [Pa(H_2_O)_10_]^5+^(H_2_O) calculated via the partial optimization by fixing the selected O-H bond distance; Figure S4: Electronic energy profile of the hydrogen transfer starting from [Pa(H_2_O)_9_(OH)]^5+^(H_2_O) calculated via partial optimization; Figure S5: Optimized geometries and key structural parameters of [PaO(H_2_O)_6_]^3+^, [PaO(H_2_O)_6_]^3+^(H_2_O) and [PaO(H_2_O)_6_]^3+^(H_2_O)’; Figure S6: Electronic energy profile of the hydrogen transfer processes starting from [Pa(OH)(H_2_O)_9_]^4+^(H_2_O) calculated via the partial optimization; Table S1: Analysis of WBI and the NBO Charges of [PaO(H_2_O)_6_]^3+^; Table S2: The reaction enthalpies and Gibbs free energies for the selected hydrogen transfer reactions obtained by different methods. Cartesian coordinates and thermal energies of all species in this study.
