# Supplementary material for: Density Functional Theory Investigations on the Mechanism of Formation of Pa(V) Ion in Hydrous Solutions
Source: Molecules. 2019 Mar 25;24(6):1169. doi: 10.3390/molecules24061169 (PMC6471942; doi:10.3390/molecules24061169)
Supplement: Supplementary file 1 [file molecules-24-01169-s001.pdf]

Supporting Information

Density Functional Theory Investigations on the Formation Mechanism of Pa(V) Ion in Hydrous Solutions

Jun Ma[a], Chuting Yang,[a] Jun Han,[a] Jie Yu,[b] Sheng Hu,[a]

Haizhu Yu,[*b] Xinggui Long[*a]

[a] *Institute of Nuclear Physics and Chemistry, China Academy of Engineering Physics, Mianyang 621900, Sichuan, China.*

[b] *Department of Chemistry and Center for Atomic Engineering of Advanced Materials, Anhui University, Hefei 230026, China.*

Emails: Xingguil@caep.cn

yuhaizhu@ahu.edu.cn

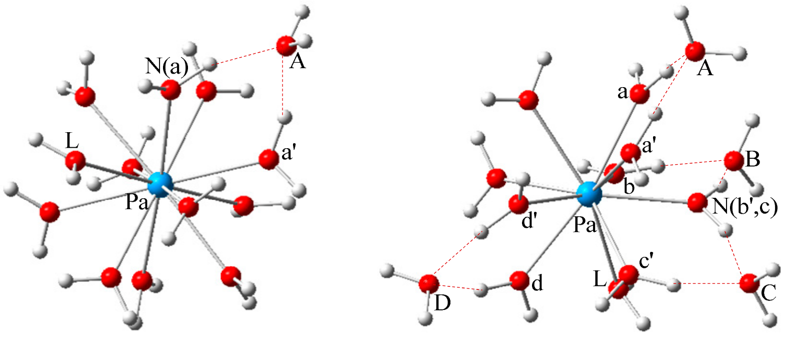

Pa-O(A):4.249, Pa-O(N):2.429
Pa-O(L):2.552,

Pa-O(A):4.218, Pa-O(B):4.091, Pa-O(C):4.187
Pa-O(D):4.269, Pa-O(N):2.337, Pa-O(L):2.564

Figure S1. Optimized geometries of $[Pa(H_2O)_n]^{5+}$ (n=13,14) gained with B3LYP/GEN/IEF-PCM method (GEN: SDD for Pa and 6-311G (d, p) for H & O atoms). The blue, red, and white balls denote Pa, O, and H atoms, respectively.

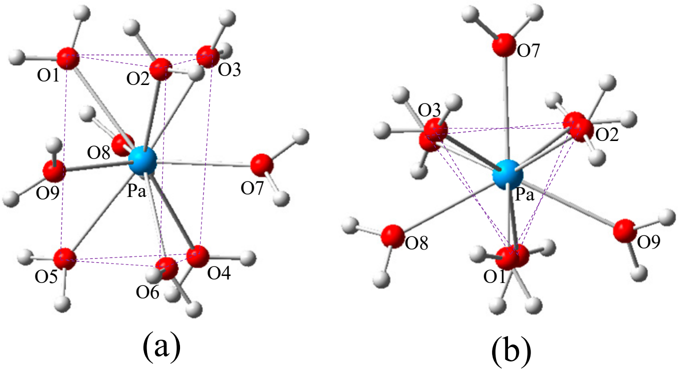

Pa-O1:0.6081, Pa-O2:0.5781, Pa-O3:0.5866, Pa-O4:0.6307, Pa-O5:0.5756
Pa-O6:0.5795, Pa-O7:0.6296, Pa-O8:0.5945, Pa-O9:0.6322

Figure S2. Pa-O Wiberg bond index (WBI) of $[Pa(H_2O)_9]^{5+}$ obtained by B3LYP/GEN/IEF-PCM method (for details see Figure S1 caption).

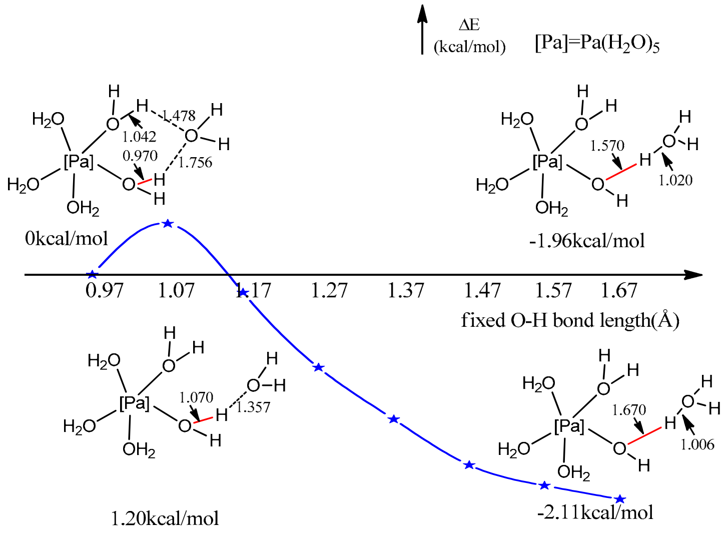

Figure S3. Electronic energy profile of the hydrogen transfer starting from $[Pa(H_2O)_{10}]^{5+}(H_2O)$ calculated via the partial optimization (by fixing the red O-H bond distance) with B3LYP/GEN/IEF-PCM method (for details see Figure S1 caption).

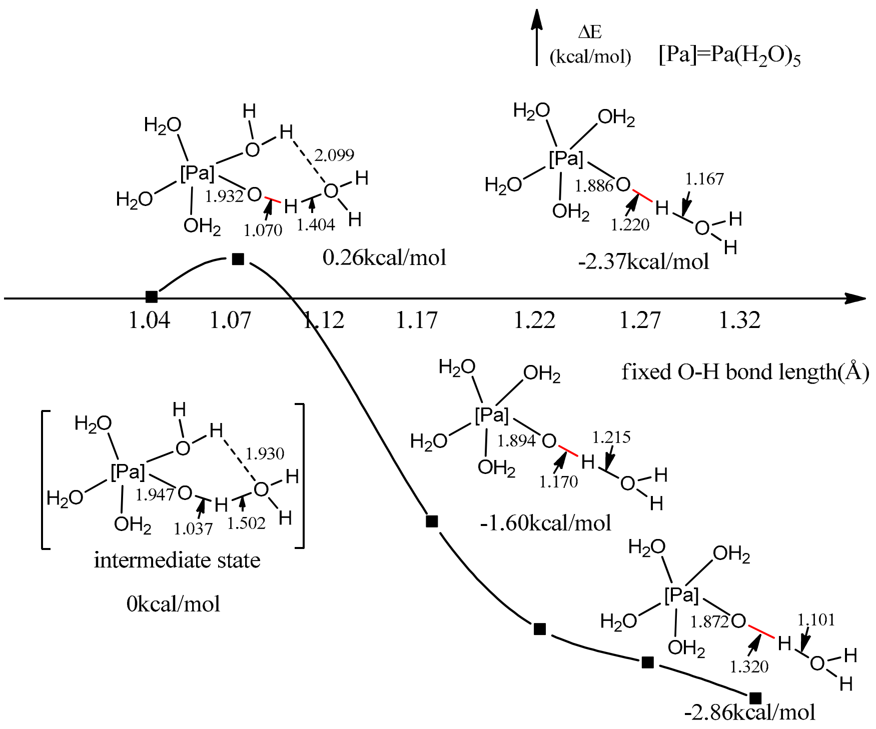

Figure S4. Electronic energy profile of the hydrogen transfer starting from $[Pa(H_2O)_9(OH)]^{5+}(H_2O)$ calculated via the partial optimization (by fixing the red O-H bond distance) with B3LYP/GEN /IEF-PCM method (for details see Figure S1 caption).

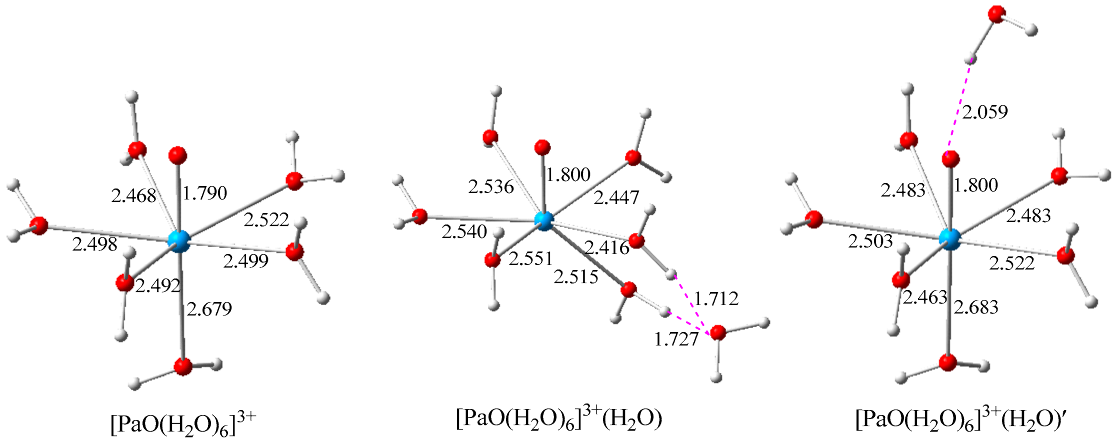

Figure S5. Optimized geometries of $[PaO(H_2O)_6]^{3+}$, $[PaO(H_2O)_6]^{3+}(H_2O)$ and $[PaO(H_2O)_6]^{3+}(H_2O)'$ obtained with B3LYP/GEN /IEF-PCM method (for details see Figure S1 caption). The blue, red, and white balls denote Pa, O, and H atoms, respectively.

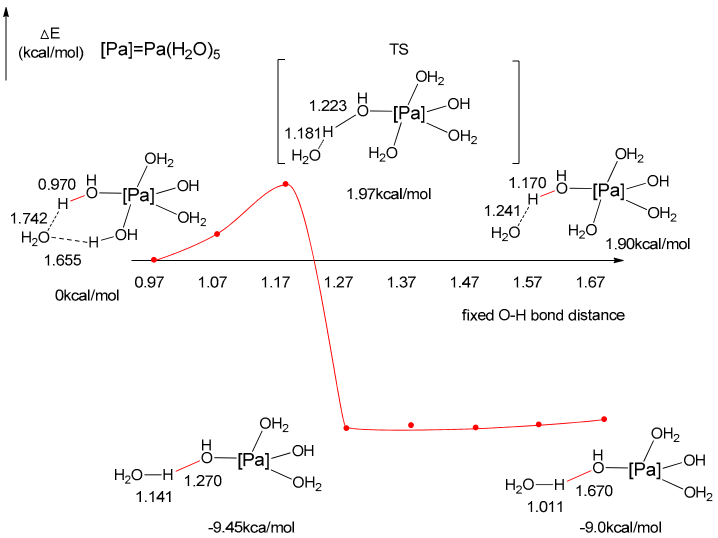

Fig S6. Electronic energy profile of the hydrogen transfer processes starting from $[Pa(OH)(H_2O)_9]^{4+}(H_2O)$ calculated via the partial optimization (by fixing the red O-H bond distance) with B3LYP/GEN/IEF-PCM method (for details see Figure S1 caption).

Table S1. Analysis of WBI and the NBO Charges of $[PaO(H_2O)_6]^{3+}$ calculated with B3LYP/GEN /IEF-PCM method (for details see Figure S1 caption).

	Oxygen in Pa=O bond	Oxygen in axial water	Oxygen in equatorial waters				
NBO	-0.808	-0.850	-0.872	-0.877	-0.877	-0.881	-0.871
WBI	2.004	1.813	1.791	1.790	1.790	1.791	1.790

Table S2. The reaction enthalpies and Gibbs free energies for the selected hydrogen transfer reactions. B3LYP-D3 method is short for: B3LYP-D3(BJ)/GEN/IEF-PCM//B3LYP/GEN/IEF-PCM (for details see Figure S1 caption) level of theory; and DKH is short for B3LYP-DKH/GEN/ IEF-PCM//B3LYP/GEN/ IEF-PCM(for details see Figure S1 caption).

**reaction**	**B3LYP-D3**		**DKH**	
	ΔH_n (kcal/mol)	ΔG_n (kcal/mol)	ΔH_f (kcal/mol)	ΔG_f (kcal/mol)
$[Pa(H_2O)_9]^{5+}\rightarrow[PaO(H_2O)_5]^{3+}(H_2O)+2H_3O^+$	-22.4	-48.4	-23.5	-49.5
$[Pa(H_2O)_9]^{5+}+H_2O\rightarrow[PaO(H_2O)_6]^{3+}(H_2O)+2H_3O^+$	-42.0	-58.2	-56.4	-72.6
$[Pa(H_2O)_9]^{5+}+2H_2O\rightarrow[PaO(H_2O)_7]^{3+}(H_2O)+2H_3O^+$	-46.3	-50.9	-62.8	-67.4
$[Pa(H_2O)_9]^{5+}+3H_2O\rightarrow[PaO(H_2O)_8]^{3+}(H_2O)+2H_3O^+$	-61.6	-56.9	-70.9	-66.1
$[Pa(H_2O)_9]^{5+}+4H_2O\rightarrow[PaO(H_2O)_9]^{3+}(H_2O)+2H_3O^+$	-71.0	-57.2	-70.7	-56.9

Cartesian coordinates and thermal energies of all species in this study:

$[Pa(H_2O)]^{5+}$

Sum of electronic and thermal Free Energies= -517.065872

Pa	0.23340600	-0.00000100	0.00000100
O	-2.01245300	-0.00002300	-0.00000700
H	-2.57043800	-0.81929900	0.00000200
H	-2.56990700	0.81960200	0.00000200

$[Pa(H_2O)_2]^{5+}$

Sum of electronic and thermal Free Energies= -593.534965

Pa	-0.00140500	-0.07151300	0.01060400
O	-2.29059000	0.24125300	0.00232000
O	2.29180500	0.23989700	0.01913200
H	2.93013600	-0.39804500	0.40798400
H	2.74718900	0.95627500	-0.47618300
H	-2.78296600	0.69691200	0.72054900
H	-2.89669900	-0.14871000	-0.66583700

$[Pa(H_2O)_3]^{5+}$

Sum of electronic and thermal Free Energies= -669.994457

Pa	0.10325100	-1.19320500	0.00627300
O	-0.23414800	-3.48988900	0.00375500
O	-1.63600600	0.33797100	-0.00007000
O	2.27890600	-0.13316100	0.00716100
H	0.45906600	-4.15380500	0.20085000
H	-1.09095800	-3.92453600	-0.18950000
H	-1.51697700	1.30540900	-0.10465300
H	-2.58206600	0.10765700	0.10875100
H	3.11289500	-0.60383200	-0.18364200
H	2.43054000	0.82023900	0.15107500

$[Pa(H_2O)_4]^{5+}$

Sum of electronic and thermal Free Energies= -746.448905

Pa	-0.00553600	0.02819200	0.00170500
O	0.71392600	2.24341300	-0.00192700
O	-2.15307600	-0.43716100	-0.81061900
O	-0.03480600	-0.66382700	2.24148400
O	1.64493000	-1.25456900	-1.04042000
H	1.22316400	2.63433900	-0.74072600
H	0.36869300	2.94257900	0.58794300
H	-2.67128000	0.15094300	-1.39385100
H	-2.70688100	-1.17147100	-0.47817600
H	0.63851500	-1.26066700	2.62421700
H	-0.49312700	-0.16727900	2.94854300
H	1.48692500	-2.13198400	-1.44242500
H	2.58676000	-1.00320500	-1.10939400

$[Pa(H_2O)_5]^{5+}$

Sum of electronic and thermal Free Energies= -822.914849

Pa	-0.23945200	0.07649600	-0.16178800
O	0.88789200	-1.79164000	0.56880500
O	-2.62063100	-0.13532300	-0.14533400
O	1.67238900	-0.33420300	-1.44351600
O	1.33114700	1.83467600	-0.13836800
O	0.70445300	0.50094000	1.96132100
H	1.66779200	-0.96305400	-2.19341500
H	2.46299100	0.23336900	-1.50158200
H	1.28629900	2.56684700	-0.78475400
H	1.91161500	2.08797300	0.60207900
H	-3.10221700	-0.81973100	0.35514200
H	-3.22934800	0.57636400	-0.41729500
H	1.78322300	-2.08409800	0.31184400
H	0.44727200	-2.49762500	1.08580400
H	1.23198500	-0.12975400	2.48463100
H	0.32537900	1.17284600	2.56209100

$[Pa(H_2O)_6]^{5+}$

Sum of electronic and thermal Free Energies= -899.369927

Pa	0.01267200	-0.03335800	-0.11371600
O	-1.38038700	-1.69465800	0.79313400
O	0.82651000	-2.21393400	-0.58083700
O	-2.29426000	0.27160100	-0.64754900
O	1.33943400	1.94109300	-0.43607800
O	-0.94651600	1.97255400	0.62852400
O	2.29747200	-0.28583200	0.56062500
H	-1.90723900	-1.54677000	1.60304400
H	-1.33806700	-2.65055900	0.60942400
H	0.72844000	-2.62547400	-1.46073300
H	1.36740000	-2.79067500	-0.01148500
H	-2.90775500	-0.48332200	-0.70141400
H	-2.58453200	0.94948100	-1.28787600

H	1.14142400	2.59592800	-1.13256300
H	2.29604500	1.95367700	-0.25784200
H	-1.84858100	2.03609500	0.99506900
H	-0.40340400	2.69797800	0.98970400
H	2.57268000	-0.26651900	1.49665600
H	3.02926600	-0.61752800	0.00852700

[Pa(H_2O)$_7$]$^{5+}$

Sum of electronic and thermal Free Energies= -975.829834

Pa	-0.03326700	-0.08649900	0.00020700
O	1.89998100	0.36270200	-1.31207000
H	1.87616000	0.40518700	-2.28638300
O	1.58262900	-1.90214000	0.00444100
H	1.36813200	-2.72606800	0.47932500
O	1.91046300	0.33422600	1.29932900
H	2.01317500	1.13829000	1.83988100
O	0.58978200	2.17124100	0.01638000
H	0.10287800	2.87143100	0.48901300
O	-1.88612200	-0.61661200	1.41723700
H	-2.53260900	-1.31109400	1.19801000
O	-1.87681600	1.42734800	-0.20252600
H	-1.97317100	2.13698900	-0.86291500
O	-1.92328800	-0.98904000	-1.19308300
H	-2.56044900	-0.46749800	-1.71062100
H	-2.68716100	1.36973200	0.33314300
H	-1.84232300	-1.86970500	-1.60513600
H	-1.82383800	-0.53042000	2.38660400
H	1.31479500	2.57630400	-0.49037600
H	2.41156400	-0.38977500	1.71623700
H	2.17167500	-2.12410300	-0.73685900
H	2.81544600	0.49034800	-1.00642900

[Pa(H_2O)$_8$]$^{5+}$

Sum of electronic and thermal Free Energies= -1052.278774

Pa	-0.01501800	-0.02471900	0.00332200
O	-0.60932000	0.24255600	-2.26706600
O	0.16601700	-2.13516900	-1.00832400
O	1.35777200	1.54352300	-1.13340800
O	-0.21141300	2.09460200	1.02137900
O	0.79861900	-0.18939900	2.23033700
O	2.21818600	-0.75690500	0.06051100
O	-1.42984000	-1.52355100	1.16401400
O	-2.25793600	0.69892800	-0.08756000
H	-2.58498200	1.61384400	-0.01842700
H	-3.01408300	0.11364400	-0.28344000
H	-1.46913700	0.56492800	-2.59178000
H	-0.13581800	-0.16805800	-3.01475600
H	-0.64467000	2.27755100	1.87645800
H	0.00152300	2.94518200	0.59566700
H	1.11433100	1.96593600	-1.97644400
H	2.18700900	1.94111700	-0.81181800
H	1.32793100	0.49853600	2.67541200
H	0.60562000	-0.89466700	2.87324700
H	2.71028000	-1.00295600	0.86404300
H	2.83479400	-0.76116900	-0.69552600
H	-0.50139100	-2.57961200	-1.56328700
H	0.90607600	-2.75137800	-0.85516300
H	-2.06508800	-1.27955700	1.86280300
H	-1.32483400	-2.49267700	1.17447700

[Pa(H_2O)$_9$]$^{5+}$

Sum of electronic and thermal Free Energies= -1128.739302

O	0.31661300	2.02849100	1.31529300
O	-0.83749400	2.05494400	-1.11146100
H	-0.07186900	2.91113000	1.19359900
H	-0.30936500	2.73668400	-1.56301900
H	-1.77899600	2.28585400	-1.20892300
O	0.22952600	-0.12808000	-2.43270500
O	-0.32282800	-1.67755300	1.57338100
O	-1.69269000	-1.57794900	-0.63676400
O	-1.99598500	0.53383300	1.06563300
O	1.75977400	1.53528300	-0.83995300
O	0.85574600	-2.14121000	-0.72093700
O	1.92851000	-0.25363600	1.18955500
H	0.76225000	2.01001700	2.18012300
H	-0.18028800	0.46009200	-3.09184800
H	0.69190300	-0.84361300	-2.90357700
H	-0.79545200	-1.55527300	2.41562100
H	0.05922800	-2.57189700	1.56145400
H	-2.24269500	-1.56008400	-1.44166700
H	-2.09119100	1.18604000	1.78305900
H	2.14892500	2.28380200	-0.35710300
H	0.35554500	-2.84909300	-1.15953800
H	1.98605300	-0.76961500	2.01412600
H	-2.03731600	-2.27042100	-0.04838000

H	-2.87303500	0.15412600	0.87689000
H	2.28375400	1.38617800	-1.64624500
H	1.79530600	-2.39276500	-0.69981700
H	2.82370500	0.05416900	0.96026800
Pa	-0.00971200	0.05169200	-0.09419400

$[Pa(H_2O)_{10}]^{5+}$

Sum of electronic and thermal Free Energies= -1205.184873

Pa	0.01847500	-0.00425600	-0.01467400
O	-1.12793500	-1.80659500	-1.19970300
O	-2.05616600	-0.83638600	1.11887100
O	-2.13219800	0.56065400	-1.09443700
O	-0.16167600	2.18860600	-1.11485200
O	-1.37331700	1.65149700	1.14052800
O	1.18272400	1.85811100	1.08269400
O	1.98885700	0.78187100	-1.18583900
O	2.09951600	-0.52099000	1.16661800
O	1.44477600	-1.71326000	-1.07327300
O	0.12966600	-2.12871800	1.15613800
H	-2.41110200	-0.47341200	1.94528100
H	-2.52419400	-1.66345600	0.92617400
H	-0.40095100	-2.33267900	1.94301900
H	0.77800700	-2.83950900	1.02277700
H	-2.19089600	1.05532500	-1.92777700
H	-2.96050500	0.07023300	-0.97192300
H	-2.32082300	1.77104700	0.96726700
H	-1.15439500	2.07517000	1.98657600
H	-1.55619700	-1.68750100	-2.06356700
H	-1.00487900	-2.75878600	-1.05596100
H	-0.83231300	2.86092300	-0.91178000
H	0.25204400	2.41356400	-1.96419100
H	1.00860000	2.79101600	0.88249200
H	1.62558800	1.80899800	1.94593000
H	2.44580000	1.62255400	-1.02198600
H	2.36780200	0.36669900	-1.97748000
H	1.25421800	-2.17659200	-1.90430400
H	2.37870900	-1.85459400	-0.85058200
H	2.95702400	-0.08416800	1.03963100
H	2.12760200	-1.01159600	2.00412300

$[Pa(H_2O)_{11}]^{5+}$

Sum of electronic and thermal Free Energies= -1281.633269

Pa	0.07320200	-0.00872400	0.12642700
O	1.22812000	-0.22671700	2.25709400
O	-0.79320600	-1.73750400	1.61843900
O	1.41539700	-2.07385300	0.28248800
O	2.50133800	0.34101700	-0.01904100
O	-1.21382100	0.83335300	2.05138300
O	0.93302900	2.07867700	1.10533300
O	-1.36267900	2.05664300	-0.25427900
O	0.83853900	1.65031500	-1.51206800
O	-2.38823700	-0.28620800	0.00001900
O	1.13599600	-0.83693300	-1.96987800
O	-0.87981000	-2.08315000	-0.97253300
H	1.25759600	-1.00735800	2.83286400
H	1.70984400	0.48509900	2.70598700
H	2.95867000	0.92433400	-0.64485700
H	3.16657700	-0.14940200	0.48908000
H	-1.19341200	-1.60436600	2.49202300
H	-1.05406700	-2.61521800	1.29813500
H	2.04675300	-2.33290900	-0.40655200
H	1.53814200	-2.67281300	1.03514200
H	-2.17747800	0.74367000	2.12442900
H	-0.86272800	1.05248800	2.92928000
H	0.36917000	2.82223500	1.36978000
H	1.85652400	2.37457600	1.14648000
H	-1.67814000	-2.08768200	-1.52353700
H	-0.37355400	-2.88601800	-1.16813000
H	0.61839800	-1.25184900	-2.67787600
H	1.99757500	-0.59158600	-2.34116500
H	-2.94926200	0.28083700	-0.55172600
H	-2.90843200	-1.05838900	0.27088000
H	-1.50301300	2.47967200	-1.11536300
H	-1.98950200	2.45074400	0.37068600
H	0.90796300	2.60098400	-1.33155200
H	0.76666300	1.52675600	-2.47264400

$[Pa(H_2O)_{12}]^{5+}$

Sum of electronic and thermal Free Energies= -1358.079117

Pa	-0.00484700	0.00149900	0.00408600
O	1.50531100	-0.10635800	1.97676300
O	-0.07498100	-2.05884400	1.40646100
O	1.94365100	-1.57270100	-0.19520700
O	2.24236400	1.00826700	-0.32623600
O	-1.04305900	0.23275100	2.24420800

O	0.37355700	2.15604100	1.22847900
O	-1.93835500	1.57694400	0.18751700
O	0.14484100	2.06001600	-1.38352300
O	-2.25617100	-1.08951700	0.28327800
O	-1.59289700	0.13567300	-1.91106400
O	1.04507700	-0.26001800	-2.23868700
O	-0.48360200	-2.12596500	-1.21736000
H	1.71136200	-0.88603600	2.51392400
H	2.09386300	0.61257500	2.25375000
H	2.46703000	1.88810300	-0.66521700
H	3.06388600	0.55712700	-0.07952900
H	-0.45526000	-2.17268100	2.29065700
H	0.12573000	-2.93876300	1.05171900
H	2.32662400	-1.90286200	-1.02143000
H	2.43179200	-1.99088600	0.52879900
H	-1.99131300	0.37701100	2.38758000
H	-0.59926700	0.20559200	3.10548200
H	0.09473900	2.31258900	2.14311200
H	0.98516800	2.86952100	0.99274300
H	-1.29519600	-2.63564100	-1.07330400
H	0.10722200	-2.65278000	-1.77482300
H	0.59763200	-0.49965500	-3.06455500
H	1.95232000	0.00671900	-2.45427900
H	-3.10038400	-0.74830100	-0.04691100
H	-2.46458500	-1.74791500	0.96277600
H	-1.97109000	-0.63212500	-2.36654400
H	-1.68809700	0.90276800	-2.49549700
H	-2.70330800	1.61034500	-0.40585700
H	-1.95785600	2.37583500	0.73528900
H	-0.33854600	2.88286600	-1.21309300
H	0.52531900	2.11369600	-2.27339000

$[Pa(H_2O)_{13}]^{5+}$

Sum of electronic and thermal Free Energies= -1434.549783

Pa	-0.18322600	-0.01236400	-0.00030200
O	-0.31286400	-1.84088900	1.77497400
O	-0.07071300	0.67123400	2.40115300
O	1.83258300	-0.56445100	1.23803800
O	0.75912500	-2.24913000	-0.62665100
O	4.06429600	0.10109500	0.02800000
O	1.35233400	1.88492600	0.50804800
O	1.84679100	0.07771500	-1.34168200
O	-0.03143300	1.80609400	-1.71603400

O	-0.42030900	-0.73199200	-2.38120100
O	-1.23351300	2.20776700	0.52835400
O	-2.36649200	0.58998400	-1.14019700
O	-2.30572800	-0.06363900	1.33000100
O	-1.77415700	-1.89127600	-0.45931200
H	1.74575900	2.09756600	1.36686300
H	1.71772300	2.49177600	-0.15188400
H	-0.53898000	2.63044000	-1.74205400
H	0.66749800	1.86400600	-2.38383900
H	4.70847800	-0.59901600	-0.15049800
H	1.91496100	-0.29313600	-2.23308000
H	2.77432200	0.18143600	-0.97715400
H	-0.81822600	-0.18608500	-3.07612000
H	-2.93809100	-0.04489800	-1.59562000
H	-2.60026000	1.46929200	-1.47032800
H	0.24726000	-3.06528300	-0.72338400
H	1.69495600	-2.46990700	-0.74572600
H	-2.37025100	-2.22983600	0.22549300
H	-2.01838300	-2.29583300	-1.30528500
H	1.84844300	-1.39392800	1.73850200
H	2.76571400	-0.31905200	0.98054800
H	0.70930300	0.48015000	2.94388800
H	-0.54402500	1.41296800	2.80515200
H	-0.58341100	-1.71904900	2.69683300
H	-0.24423500	-2.79466100	1.62412700
H	-2.11876900	2.34017000	0.89942300
H	-0.69699700	2.98408800	0.74719700
H	4.56798500	0.90392300	0.22294900
H	-0.04877700	-1.52731300	-2.79052400
H	-3.16949300	0.07076500	0.91363500
H	-2.43841700	-0.17298000	2.28258100

$[Pa(H_2O)_{14}]^{5+}$

Sum of electronic and thermal Free Energies= -1511.071117

Pa	0.21029100	-0.12100600	-0.18840700
O	0.22675600	1.71959500	1.30840100
O	-2.09196400	2.80401700	1.72903100
O	2.24741400	1.10067700	-0.49429800
O	0.18412500	2.09874100	-1.47051700
O	-1.89545800	0.88365700	-0.05975000
O	-0.73995100	-0.20490000	-2.37741200
O	-3.24617200	0.46489700	-2.29714400
O	-1.61165300	-1.73113200	-0.42165900

O	-2.76435200	-2.29744700	1.86264500
O	1.74654300	-1.05972400	-1.81713400
O	0.75612900	-2.50186800	0.11005300
O	-0.66888900	-0.75546200	1.93220800
O	4.29224100	0.37852200	0.95682500
O	1.92201400	-0.56808300	1.41610700
H	-3.94666700	-0.19611000	-2.39167800
H	-3.54018700	1.25315500	-2.77585500
H	-2.58862300	0.75606600	-0.76337900
H	-2.17403100	1.61935200	0.55268300
H	-2.56906500	2.66278600	2.55866100
H	-2.25797800	3.71727500	1.45611500
H	-0.14575900	2.11652400	-2.38003800
H	-0.00460900	2.96369100	-1.07748500
H	-1.70209500	-0.00312900	-2.58222700
H	-0.28352600	-0.52988600	-3.16833500
H	2.21775600	1.90926000	-1.02881300
H	3.15406200	0.96593300	-0.09890600
H	-0.55898100	2.26322300	1.61926200
H	1.04107500	2.09029800	1.68109000
H	1.78230900	-1.99253400	-2.07992400
H	2.46752500	-0.58046900	-2.25303800
H	4.99088800	-0.19941500	0.61924800
H	4.70713100	1.00311500	1.56820200
H	1.74505200	-1.08454000	2.21624900
H	2.88910400	-0.30011900	1.40608500
H	0.10795400	-3.21922500	0.07372200
H	1.59585600	-2.85638100	0.43715100
H	-2.23619700	-2.02468500	0.29694500
H	-1.82512700	-2.19247900	-1.24501200
H	-1.44622800	-1.37321300	2.08389400
H	-0.58110100	-0.18728000	2.71221600
H	-3.61148100	-1.91933900	2.13759300
H	-2.73882600	-3.20660800	2.19252500

$[Pa(H_2O)_{10}]^{5+}(H_2O)^F$

Sum of electronic and thermal Free Energies= -1281.658937

O	-3.70656900	0.69616200	-0.77346300
H	-4.18080300	1.15989200	-1.47959000
H	-4.35230500	0.47621400	-0.08554800
H	-2.51305100	-0.50561800	-1.23824800
H	-1.65809600	-1.74488900	-1.71000600
O	-1.62494700	-0.89570200	-1.24075900
Pa	0.22313700	-0.06303700	0.08617200
H	-1.52684800	-0.17558200	2.58306200
O	-0.69787400	0.24500300	2.29962000
H	-0.57299100	1.04525300	2.83381400
O	-1.42276000	1.50041400	-0.18134500
H	-1.28201500	2.45878900	-0.09368700
H	-2.43500200	1.30945100	-0.33731000
O	-0.89574600	-2.03525500	1.02977700
H	-0.61198900	-2.62504400	1.74616500
H	-1.77695600	-2.32210600	0.74327200
O	0.86225600	-2.14819200	-0.95722500
H	1.10678000	-2.26952200	-1.88927000
H	0.81407600	-3.02550400	-0.54300400
O	0.98441100	2.12451400	-0.77276500
H	0.72097200	2.43541400	-1.65350300
H	1.76195500	2.64170500	-0.51208300
O	0.50950000	0.08662600	-2.32122400
H	-0.22412700	0.00173400	-2.95168600
H	1.31265000	0.32215400	-2.81337700
O	1.50976300	-1.22852600	1.78366400
H	2.39152300	-1.62810300	1.70100700
H	1.22968200	-1.32373800	2.70907900
H	2.10322700	1.10875000	2.25758700
O	1.51080600	1.42707700	1.55693600
H	1.38648900	2.38120800	1.69735200
O	2.55633700	-0.19130000	-0.49188900
H	2.99834900	-0.96554700	-0.87754000
H	3.24017900	0.47808500	-0.32525500

$TS\text{-}H1^F$

Sum of electronic and thermal Free Energies= -1281.661520

O	-3.71916900	0.66966400	-0.74612200
H	-4.04295000	1.15662900	-1.51785600
H	-4.46476000	0.16102900	-0.39567200
H	-2.59476000	-0.06148300	-0.95433500
H	-1.83544400	-1.40513500	-1.54630800
O	-1.67590100	-0.60666700	-1.01241100
Pa	0.24518700	-0.07046100	0.03533900
H	-1.48466400	-0.52363300	2.51999400
O	-0.83460800	0.11990400	2.19609300
H	-0.79894500	0.86484900	2.81737400
O	-1.28805100	1.76060600	0.26327900
H	-1.26004900	2.54648500	-0.30836600

H	-2.17675700	1.71488700	0.66028300
O	-0.86439100	-2.10267300	1.02173300
H	-0.50233300	-2.71757400	1.67896300
H	-1.73117700	-2.44137500	0.74859300
O	0.80015900	-2.19159500	-0.94427000
H	0.95806900	-2.33824900	-1.89144300
H	0.65209900	-3.05286300	-0.52326000
O	1.09379800	1.98989900	-0.96027900
H	0.99512900	2.21195300	-1.89971100
H	1.55652100	2.72289700	-0.52507200
O	0.57621600	-0.11822100	-2.44880600
H	-0.14469800	-0.05150600	-3.09469700
H	1.41411200	-0.01945900	-2.92820500
O	1.54261100	-1.14474900	1.75727700
H	2.45315800	-1.47455700	1.67904900
H	1.28728000	-1.20585000	2.69302100
H	2.06815100	1.32718400	2.11605800
O	1.31592800	1.54165200	1.54107600
H	1.00734200	2.42750100	1.79174300
O	2.57495500	-0.24892700	-0.54512700
H	3.05072400	-1.05819800	-0.79676300
H	3.19310900	0.49490700	-0.63812400

$[Pa(OH)(H_2O)_9]^{4+}(H_3O)^+$

Sum of electronic and thermal Free Energies=

-1281.669287

O	-4.22481700	0.33203400	-0.56488500
H	-4.52679500	0.96400700	-1.24512200
H	-4.78419500	-0.46852800	-0.57522600
H	-3.26060800	0.10000300	-0.69068000
H	-1.84526700	-1.06391700	-1.68327400
O	-1.62715900	-0.44138400	-0.96545800
Pa	0.15873200	-0.02136800	-0.00866800
H	-1.58340900	-0.42111900	2.46818200
O	-0.91078300	0.21030900	2.16908900
H	-0.73732500	0.84509900	2.88087100
O	-1.05400700	2.06193300	0.38838400
H	-1.09309100	2.77422700	-0.26841800
H	-1.79699200	2.17196100	1.00121400
O	-1.01204500	-2.05704700	0.99293000
H	-0.67307600	-2.68037400	1.65183600
H	-1.82837100	-2.42950400	0.63023900
O	0.80459900	-2.19172200	-0.91431100
H	0.87702500	-2.35984200	-1.86654900
H	0.60044600	-3.03148000	-0.47753200
O	1.16965000	2.00683400	-1.06794900
H	1.07966100	2.22343800	-2.00726300
H	1.62339900	2.74423400	-0.63388400
O	0.57400700	-0.11333900	-2.47847200
H	-0.08624700	0.01113200	-3.17696100
H	1.44964100	-0.08174500	-2.89298600
O	1.40795500	-1.20323900	1.73744700
H	2.31729000	-1.53183400	1.67053900
H	1.16443700	-1.20871900	2.67624300
H	2.15102400	1.29015800	2.06283000
O	1.38516800	1.51310000	1.51194100
H	1.08737100	2.39830600	1.77097400
O	2.53247200	-0.25178300	-0.54167700
H	3.00389000	-1.07052000	-0.76077700
H	3.15271500	0.48801100	-0.62801700

$[Pa(OH)(H_2O)_9]^{4+}$

Sum of electronic and thermal Free Energies=

-1204.864898

H	-2.46055200	-0.44378400	-1.26075900
O	-1.57565400	-0.37610500	-0.86199300
Pa	0.17988100	-0.05332700	-0.02315300
H	-1.64291500	-0.50586600	2.39484000
O	-0.97953600	0.14401300	2.11995800
H	-0.90964800	0.83010500	2.79882300
O	-1.02063900	2.08860700	0.36765000
H	-0.97523000	2.78114000	-0.30837000
H	-1.92534700	2.08001300	0.71335900
O	-0.95742200	-2.13774300	0.94254600
H	-0.63837700	-2.75739000	1.61359800
H	-1.74915700	-2.51712700	0.53662400
O	0.95274300	-2.22832100	-0.89882700
H	0.97379300	-2.40215300	-1.85158600
H	0.69715500	-3.05257200	-0.46067100
O	1.21921000	2.00987400	-1.07311900
H	1.13457700	2.23642300	-2.01001100
H	1.70076800	2.72470500	-0.63303100
O	0.51555600	-0.11638900	-2.47317200
H	-0.22347800	-0.19556600	-3.09405300
H	1.33479700	-0.06697500	-2.98763900
O	1.42690200	-1.21081800	1.76377600
H	2.34839300	-1.50333500	1.70899900
H	1.17688100	-1.20048800	2.69992100

H	2.10241100	1.34746100	2.08810100
O	1.33288900	1.52404800	1.52739700
H	0.96769300	2.38447700	1.78192600
O	2.58562400	-0.24304800	-0.50985900
H	3.02986900	-1.06597700	-0.76401500
H	3.19067400	0.49595200	-0.66813800

$[PaO(H_2O)_9]^{3+}(H_3O)^+$

Sum of electronic and thermal Free Energies=

-1281.338023

O	3.87535600	-1.04194800	-0.07250600
H	4.16977600	-1.11144700	-0.99637300
H	4.46916300	-0.43726300	0.40353600
H	2.84791800	-0.72898100	-0.00295600
O	1.51940600	-0.38929300	0.09847000
Pa	-0.32851900	-0.14236000	0.05230700
H	-0.00143200	-2.29705500	2.25164900
O	-0.59375900	-2.13619400	1.50482700
H	-1.11350200	-2.93584500	1.34814600
O	-0.09954200	-2.36178900	-1.12999200
H	-0.02188300	-2.32956500	-2.09399700
H	0.52439000	-3.02843300	-0.81122500
O	0.58036400	0.00037700	2.53508100
H	0.11302500	0.06229700	3.37867900
H	1.52207000	-0.09896000	2.72746600
O	0.03610600	2.13422200	1.03444500
H	0.58799100	2.77240200	0.56100500
H	0.34227200	2.11410700	1.95290500
O	-1.14314600	-0.27767000	-2.38049500
H	0.70117200	0.16821400	-3.11466000
H	-1.99150800	-0.61606700	-2.69559700
O	0.63669400	1.52105700	-1.57053400
H	1.58232100	1.56429500	-1.76649800
H	0.20332300	2.26331200	-2.01372200
O	-2.05017700	0.23762900	1.83923600
H	-2.68937300	0.96179000	1.87430400
H	-2.31696800	-0.42782400	2.48821300
H	-3.36832400	-1.17460800	0.06473800
O	-2.52583700	-1.35019500	-0.37551300
H	-2.58000800	-2.22491000	-0.78512000
O	-2.02259900	1.62320800	-0.54222400
H	-2.02728500	2.51190700	-0.15989400
H	-2.67739800	1.59417600	-1.25240200

$[PaO(H_2O)_9]^{3+}$

Sum of electronic and thermal Free Energies=

-1204.500502

O	1.53288300	-0.04555100	-0.12341900
Pa	-0.27250500	0.00695700	-0.04160600
H	0.40034200	-2.07845200	2.12222500
O	-0.18434200	-2.06266500	1.35287600
H	-0.73306500	-2.85705000	1.36656300
O	0.07351600	-2.18602500	-1.33574800
H	0.21864400	-2.13087200	-2.28987900
H	0.74993500	-2.78086300	-0.98485500
O	0.79385100	0.18621900	2.41455800
H	0.43410700	0.24650700	3.30912100
H	1.75589200	0.23734500	2.48185600
O	-0.14533800	2.31995900	0.99730600
H	0.36119300	2.96394700	0.48289600
H	0.21851400	2.32022800	1.89361400
O	-1.30462900	-0.06323400	-2.40670500
H	-0.89580400	0.29682900	-3.20411800
H	-2.04448900	-0.62396200	-2.67324000
O	0.42136900	1.89944800	-1.61593400
H	1.34491300	1.94594300	-1.89564000
H	-0.07480700	2.54998000	-2.12997500
O	-1.92922100	0.16405600	1.88950500
H	-2.70454700	0.74064300	1.85828100
H	-2.10021300	-0.52241700	2.54806700
H	-3.20874000	-1.40884000	0.08484700
O	-2.37530600	-1.44824500	-0.40127600
H	-2.29365500	-2.33368000	-0.78153000
O	-2.28349800	1.54479700	-0.40769600
H	-2.27992600	2.45178700	-0.07225200
H	-2.80396100	1.52604200	-1.22175200

$[PaO(H_2O)_9]^{3+}(H_2O)$

Sum of electronic and thermal Free Energies=

-1280.958328

O	2.12248400	-3.21522100	0.74748800
H	2.16309200	-4.17362600	0.86090300
H	3.02486500	-2.89047300	0.86319900
O	1.56395400	0.19404800	0.25711700
Pa	-0.23717100	0.06969500	0.09513400
H	0.82065700	-2.38529500	1.55312100
O	-0.01350000	-1.86043300	1.62490300
H	-0.72369300	-2.48371300	1.82565600

O	0.47799800	-2.06992900	-1.09204400
H	0.74036000	-1.97763300	-2.01693800
H	1.18180900	-2.58014200	-0.63189600
O	0.58267300	0.70637800	2.73720600
H	0.36438500	0.16898900	3.50911400
H	1.54779800	0.71135800	2.68981900
O	-0.54928900	2.37461800	1.11453000
H	-0.06717500	3.09891200	0.69311600
H	-0.24019800	2.30931300	2.03577400
O	-0.93934100	-0.13849800	-2.37035100
H	-0.52148100	0.30440800	-3.11928600
H	-1.58190900	-0.77473300	-2.70973000
O	0.42645100	2.02489600	-1.41413900
H	1.36556700	2.19994200	-1.55720400
H	-0.07422700	2.62738100	-1.97959700
O	-2.08841900	0.00336100	1.84250500
H	-2.91937500	0.47723800	1.70661200
H	-2.11236900	-0.37973300	2.72829000
H	-2.95290900	-1.70041100	-0.02785100
O	-2.10708500	-1.60776900	-0.48521500
H	-1.80888600	-2.48920200	-0.75070500
O	-2.41175500	1.29298500	-0.48172900
H	-2.53243600	2.20185800	-0.17332800
H	-2.84019600	1.20966100	-1.34387600

$[Pa(H_2O)_6]^{5+}(H_2O)^F$

Sum of electronic and thermal Free Energies=

-975.832714

Pa	0.11254300	-0.04403400	-0.19984500
O	-1.28487300	-1.81160300	0.64184700
O	0.97054000	-2.07762700	-0.73706300
O	-2.21177600	0.23653700	-0.76800700
O	1.39713500	1.92130000	-0.40543900
O	-0.96968700	1.94986400	0.60786900
O	2.30850700	-0.29469800	0.62222100
H	-1.80923300	-1.70721400	1.45753500
H	-1.16911000	-2.76042700	0.46541000
H	0.73001700	-2.52746800	-1.56210300
H	1.55386600	-2.63189900	-0.18691600
H	-2.80232600	-0.53571200	-0.82276100
H	-2.50630100	0.89704700	-1.42185400
H	1.16695100	2.63632700	-1.02891700
H	2.33718400	2.00166600	-0.16821300
H	-1.89001800	1.93412600	0.92565300
H	-0.48331000	2.64320300	1.08835000
H	2.53710000	-0.22098100	1.56834000
H	3.10011200	-0.55614400	0.11581000
O	0.20796700	-3.08930100	-2.84146900
H	0.85957100	-3.26097500	-3.53374300
H	-0.31672100	-3.89643600	-2.75878200

$[Pa(OH)(H_2O)_5]^{4+}(H_3O)^+$

Sum of electronic and thermal Free Energies=

-975.864624

Pa	-0.04574100	-0.07626200	0.05842700
O	-1.57141200	-1.60470600	1.18400800
O	0.50311500	-1.93068300	-0.48006400
O	-2.38033800	0.21361200	-0.53421600
O	1.43654200	1.80307400	-0.50799900
O	-0.91118600	2.11817900	0.53490400
O	2.29773900	-0.35493500	0.62613300
H	-1.91805400	-1.40317400	2.07039300
H	-1.57565100	-2.56813900	1.07294900
H	0.59541800	-2.39136400	-2.45390300
H	0.88276100	-2.75879800	-0.12309800
H	-3.03369700	-0.45668800	-0.27526300
H	-2.68677600	0.64876900	-1.34806800
H	1.13076000	2.53433500	-1.07036200
H	2.38269200	1.66880800	-0.67416400
H	-1.86382100	2.24709200	0.67985200
H	-0.42207000	2.79434500	1.03419000
H	2.70853500	0.06755700	1.39887200
H	2.88622000	-1.05895300	0.31372600
O	0.61928700	-2.67041400	-3.39686300
H	1.52640100	-2.60538500	-3.75574500
H	0.27613900	-3.58069900	-3.49663900

$[Pa(OH)(H_2O)_5]^{4+}$

Sum of electronic and thermal Free Energies=

-899.055059

Pa	0.04478600	-0.02988400	0.00066700
O	-1.34946300	-1.70643500	0.98955900
O	0.71146300	-1.73961500	-0.66346200
O	-2.29231800	0.17949300	-0.65486800
O	1.43411800	1.93578400	-0.33664800
O	-0.98802300	2.16816100	0.49929500
O	2.37690400	-0.25597800	0.69644400
H	-1.87011900	-1.55966300	1.79741700

H	-1.20776800	-2.65991200	0.88299000
H	1.03405600	-2.55046000	-1.10215500
H	-2.92093500	-0.54701900	-0.51718100
H	-2.57280900	0.67417600	-1.44390500
H	1.15497300	2.71199700	-0.84910100
H	2.39389900	1.83220200	-0.44108100
H	-1.94522300	2.21862800	0.65201300
H	-0.55572600	2.80628500	1.09009400
H	2.71136200	0.12722400	1.52432200
H	2.85661500	-1.08281900	0.53809700

$[PaO(H_2O)_5]^{3+}(H_3O)^+$

Sum of electronic and thermal Free Energies=

-975.536980

Pa	0.02768700	-0.21879200	-0.01112600
O	-1.54733000	-1.74512500	1.12285400
O	0.68714400	-1.89450000	-0.36610200
O	-2.37226600	0.15517400	-0.66755700
O	1.54283100	1.71611800	-0.57165700
O	-0.86378500	2.01336800	0.44903000
O	2.28540700	-0.26420900	0.99884200
H	-2.08430300	-1.50928000	1.89424200
H	-1.37238700	-2.69570700	1.16278800
H	1.00220400	-3.30118100	-0.80443700
H	-3.00439200	-0.56986600	-0.55499000
H	-2.61886500	0.63504000	-1.47283400
H	1.29984800	2.38800600	-1.22582800
H	2.49410000	1.55641800	-0.65492700
H	-1.80609200	2.14749500	0.63293200
H	-0.35294300	2.71249100	0.88510100
H	2.56852900	0.30240700	1.73195800
H	2.75360100	-1.10690800	1.07877800
O	1.15880000	-4.28171500	-1.07963600
H	1.27882500	-4.36598500	-2.04258900
H	1.92902500	-4.66168800	-0.62002700

$[PaO(H_2O)_5]^{3+}$

Sum of electronic and thermal Free Energies=

-898.724727

O	-2.37315900	0.69741600	0.06534700
H	-2.66995600	1.59669800	0.25561500
H	-3.00549200	0.07546000	0.44819900
O	1.64327100	-1.84320400	0.23103800
O	-1.54427100	-1.88759700	0.34373200
O	-0.01899100	2.37055400	0.40227100
O	2.34446500	0.79780800	0.10890200
H	1.64168900	-2.25788800	1.10425500
H	1.92878000	-2.51061100	-0.40847000
H	-1.52851400	-2.25596400	1.23696700
H	-0.07368300	2.65522800	1.32411400
H	2.97275400	0.14151300	0.44007200
H	-1.86959700	-2.57257100	-0.25677000
H	-0.01346600	3.15624400	-0.16264700
H	2.53441700	1.63955500	0.54329800
Pa	-0.00483400	-0.00144200	-0.28330700
O	0.01405200	-0.07703300	1.50574400

$[PaO(H_2O)_5]^{3+}(H_2O)$

Sum of electronic and thermal Free Energies=

-975.169045

Pa	0.22089100	-0.05175500	-0.41563100
O	-0.78093000	-2.27914500	-0.54606900
O	1.45514300	-0.87130900	-1.42748200
O	-2.19578400	0.08096900	-1.07034300
O	1.57800600	2.04968600	-0.41925600
O	-1.00651600	1.90509000	0.51205800
O	2.38231300	-0.02930900	0.99229700
H	-1.74332900	-2.50360900	-0.53941700
H	-0.27799200	-3.03608400	-0.87158500
H	-2.81329300	-0.69081600	-1.04017700
H	-2.51947100	0.70882000	-1.73035700
H	1.53142000	2.73751100	-1.09799700
H	2.50099400	1.94960500	-0.14612000
H	-1.95509800	1.90626000	0.69938400
H	-0.58730400	2.64584700	0.97136000
H	2.36660400	0.10145100	1.95098300
H	3.01947100	-0.73317100	0.81157100
O	-3.42834200	-2.27039700	-0.75237100
H	-3.82863600	-2.73599700	-1.49856500
H	-4.02317000	-2.39201900	-0.00077300

$[Pa(H_2O)_7]^{5+}(H_2O)^F$

Sum of electronic and thermal Free Energies=

-1052.294202

Pa	-0.28459300	0.00879600	0.40988600
O	1.55638200	0.23112900	-0.88257200
H	1.47910200	0.33796300	-1.85234700
O	0.95380000	-2.00581300	0.42426700

H	0.69193400	-2.78379300	0.95289900
O	1.63673200	0.20047000	1.79724000
H	1.91557800	1.01523900	2.24934100
O	0.41925700	2.29117600	0.71716800
H	-0.06809100	2.88777900	1.31450400
O	-2.61163700	-0.69544100	0.73284200
H	-2.97639500	-1.49508600	0.31685700
O	-1.94132800	1.23767500	-0.81763800
H	-1.78329900	2.00590700	-1.39479300
O	-0.99153100	-1.28021600	-1.41004800
H	-1.54034500	-0.94509300	-2.14374000
H	-2.88422900	1.19522700	-0.58205700
H	-0.72017800	-2.19362200	-1.61430300
H	-3.02190100	-0.60045400	1.61105200
H	0.98441100	2.82770500	0.13486400
H	2.13742400	-0.55027200	2.16092500
H	1.70854100	-2.24994100	-0.14164500
H	2.48415900	0.37509000	-0.63884100
O	3.91156300	0.58156500	-0.31265000
H	4.51805800	0.03639000	-0.83124100
H	4.22165000	1.49214800	-0.40575700

$[Pa(OH)(H_2O)_6]^{4+}(H_3O)^+$

Sum of electronic and thermal Free Energies=

-1052.320978

Pa	-0.20819700	0.02732100	0.27718900
O	1.49152400	0.14627800	-0.83704900
H	1.56605300	0.20659400	-1.81203200
O	0.88987800	-2.13143800	0.24325400
H	0.60899400	-2.90884900	0.75513500
O	1.45575400	0.05498600	2.00933500
H	1.67082100	0.83613400	2.54452700
O	0.51144100	2.32227800	0.57586500
H	0.00571600	2.97346500	1.09054800
O	-2.57630300	-0.58904700	0.86591600
H	-2.88960300	-1.49590300	0.71075600
O	-1.99287600	1.18474700	-0.91940900
H	-1.89070500	1.92614000	-1.53853400
O	-1.06447800	-1.28852200	-1.48020800
H	-1.69934000	-0.98115900	-2.14887000
H	-2.91809900	1.12096300	-0.63295700
H	-0.74847900	-2.17562500	-1.72051200
H	-2.90114600	-0.31910100	1.74174600
H	1.20231000	2.79027700	0.08024200
H	1.80864300	-0.72985700	2.45846700
H	1.73870700	-2.32502500	-0.18512700
H	3.23187900	0.49709000	-0.43823400
O	4.17284000	0.78914500	-0.32270500
H	4.76356700	0.32348000	-0.94619600
H	4.25230600	1.75662800	-0.43414800

$[Pa(OH)(H_2O)_6]^{4+}$

Sum of electronic and thermal Free Energies=

-975.525340

Pa	-0.30751600	-0.06030400	0.56497900
O	0.94082400	0.38172600	-0.85488800
H	1.53199000	0.57247600	-1.60699600
O	0.97335400	-2.10139600	0.15960800
H	0.97564200	-2.81833800	0.81481600
O	1.77359200	0.17661600	1.73068300
H	2.67006400	0.26329200	1.37364800
O	0.15099500	2.30449000	0.80672000
H	-0.36970000	2.89933800	1.36801800
O	-2.78257600	-0.59640600	0.71642000
H	-3.13394300	-1.40614000	0.31789800
O	-1.69427900	1.14893400	-0.97062200
H	-1.38393100	1.84796600	-1.56628400
O	-1.16270400	-1.46130100	-1.21559600
H	-1.57584100	-1.10395600	-2.01616400
H	-2.65474000	1.22626100	-0.85760800
H	-0.71983300	-2.29544100	-1.43413900
H	-3.30614600	-0.39245200	1.50594700
H	0.97521200	2.75084700	0.56473800
H	1.82606200	0.17850700	2.69994200
H	1.81060200	-2.13825000	-0.32602400

$[PaO(H_2O)_6]^{3+}(H_3O)^+$

Sum of electronic and thermal Free Energies=

-1052.000987

Pa	-0.19440600	-0.08864800	0.72010400
O	1.25056400	0.37506800	-0.31938400
O	1.04782800	-2.16916200	0.77764900
H	0.79455500	-2.96802200	1.26431900
O	1.62926600	0.08102500	2.43418200
H	1.78529100	0.93567900	2.85878000
O	0.25137300	2.25397400	1.38897400
H	-0.34899600	2.79552600	1.92165100
O	-2.67626000	-0.50369300	0.39246800

H	-2.96311600	-1.23309100	-0.17589500
O	-1.22907500	1.23977500	-1.04937000
H	-0.78435900	1.91239400	-1.58339300
O	-0.71358600	-1.38995200	-1.30987300
H	-0.83615800	-0.93314600	-2.15387200
H	-2.18065200	1.42053200	-1.05097900
H	-0.33932900	-2.26366200	-1.49023800
H	-3.32379100	-0.40794300	1.10604400
H	0.83270400	2.84842400	0.89514100
H	1.81953500	-0.60920200	3.08444000
H	1.93604100	-2.30061400	0.41648500
H	2.52236000	0.52207300	-1.00981600
O	3.46464700	0.58314900	-1.45842200
H	3.50261100	0.06428000	-2.28115800
H	3.71366600	1.50515800	-1.64521200

$[PaO(H_2O)_6]^{3+}$

Sum of electronic and thermal Free Energies=

-975.162285

O	2.34891700	0.75324700	-0.40316200
H	2.54498200	1.56211800	-0.89376900
H	3.01148100	0.09080100	-0.64264800
O	0.00726800	0.35674500	2.69809300
O	-1.54661100	-1.94354900	-0.28778900
O	1.59527600	-1.91666900	-0.32151500
O	0.00103900	2.33331800	-0.68878300
O	-2.37457700	0.68899200	-0.31826800
H	0.80333800	0.77317500	3.05857000
H	-0.74206800	0.86597200	3.03910700
H	-1.54634100	-2.47194000	-1.09700200
H	-1.84904500	-2.51213700	0.43520900
H	1.61786800	-2.41375400	-1.14994900
H	-0.03849800	2.59394700	-1.61932200
H	-2.99643000	0.00399200	-0.59899500
H	1.90745800	-2.50088100	0.38450700
H	0.00054500	3.13492700	-0.14757000
H	-2.62051000	1.51170100	-0.76116700
Pa	0.01697800	-0.02312400	0.04592500
O	-0.01415800	-0.21819800	-1.73345800

$[PaO(H_2O)_6]^{3+}(H_2O)$

Sum of electronic and thermal Free Energies=

-1051.631468

Pa	-0.15480700	0.02415400	0.66456500
O	0.91245200	0.96289700	-0.43955000
O	1.96766400	-1.38847300	0.57148000
H	1.97944000	-2.33821600	0.75337300
O	1.58184600	0.51153100	2.45301300
H	1.70314800	1.42852100	2.73165700
O	-0.22017600	2.36448300	1.63890600
H	-0.92627100	2.67480900	2.22269200
O	-2.42976200	-1.04585700	0.57894400
H	-2.63968900	-1.77575600	-0.05536800
O	-1.81310800	1.27131200	-0.63192400
H	-1.63752600	1.95275100	-1.29288600
O	-0.38053900	-1.26244200	-1.36804100
H	0.06931100	-1.01263100	-2.18444800
H	-2.74895700	1.03269000	-0.66030700
H	-1.05729400	-1.95764600	-1.56024300
H	-2.90953900	-1.21691500	1.40130000
H	0.03689000	3.10871000	1.07895000
H	2.45083600	0.10038900	2.35614500
H	2.57544100	-1.23104900	-0.16322400
O	-2.49421600	-2.87064700	-1.38339700
H	-3.14726700	-2.79780900	-2.09195100
H	-2.40738300	-3.81228400	-1.18478200

$[Pa(H_2O)_8]^{5+}(H_2O)^F$

Sum of electronic and thermal Free Energies=

-1128.743140

Pa	0.27010600	-0.01433600	0.05724900
O	-0.04245400	0.63765200	2.32053200
O	2.37297300	-0.12210000	1.16032000
O	-1.16940400	-1.58274400	1.10149700
O	0.60334300	-0.62499900	-2.21946500
O	1.18764000	-2.20243200	-0.09358100
O	1.79746000	1.47760900	-0.97500700
O	-0.59203500	2.19556800	0.20381100
H	-1.38229700	2.51732800	-0.26312800
H	-0.20127900	2.93936500	0.69718700
H	-0.80165900	1.14985900	2.65359200
H	0.45959800	0.30043200	3.08456800
H	-1.41917400	-1.59327400	2.04208900
H	-1.68970200	-2.26817200	0.64428200
H	0.24894200	-1.40982300	-2.67530200
H	1.14155700	-0.11660300	-2.85071600
H	1.86479000	-2.49024500	-0.73182800
H	0.97626200	-2.95070700	0.49174000

H	2.82294000	0.61885100	1.60582100
H	2.85474900	-0.94292600	1.36257200
H	1.57337500	2.33533800	-1.37804100
H	2.75831900	1.33878500	-1.05904100
O	-4.11049500	0.00802900	-0.30973000
H	-2.66389400	0.06741300	-0.68695300
H	-4.60452700	-0.68585500	-0.76619600
H	-4.59149200	0.82906100	-0.47771400
O	-1.74854300	0.11611400	-1.00422200
H	-1.77114200	0.25433000	-1.96903200

$[Pa(OH)(H_2O)_7]^{4+}(H_3O)^+$

Sum of electronic and thermal Free Energies=

-1128.769358

Pa	0.18940300	0.00563400	0.01480000
O	-0.11723600	0.61228500	2.31937000
O	2.33832800	-0.10933400	1.16199900
O	-1.17122600	-1.63822400	1.11674800
O	0.66667700	-0.58219100	-2.28781400
O	1.16940300	-2.20335100	-0.09234300
O	1.82676200	1.46764400	-0.97785600
O	-0.59174100	2.26400200	0.15051600
H	-1.37746500	2.60076000	-0.30879200
H	-0.16365600	3.00441400	0.61259200
H	-0.84983800	1.15598100	2.65428800
H	0.41872900	0.31756700	3.07440500
H	-1.33795800	-1.68312200	2.07182700
H	-1.73079200	-2.29911000	0.67664800
H	0.31032700	-1.33834200	-2.78162600
H	1.30217300	-0.11579200	-2.85239700
H	1.82098400	-2.51036200	-0.74374300
H	0.90996600	-2.95299300	0.46632800
H	2.78798300	0.61663300	1.62595400
H	2.80728500	-0.93791900	1.35040000
H	1.63732900	2.30649300	-1.42955200
H	2.78815800	1.32856100	-0.97558400
O	-4.23711300	-0.00106100	-0.19910100
H	-3.27677300	0.04588800	-0.46878600
H	-4.68445100	-0.75765200	-0.62449600
H	-4.70373200	0.83029100	-0.41131400
O	-1.60902800	0.09259100	-0.99264300
H	-1.72119300	0.24142900	-1.95124700

$[Pa(OH)(H_2O)_7]^{4+}$

Sum of electronic and thermal Free Energies=

-1051.959403

Pa	0.17367000	0.01395700	-0.01200200
O	-0.10418600	0.64618800	2.32449600
O	2.34351600	-0.09761400	1.17090300
O	-1.14665200	-1.69492400	1.11687800
O	0.67585700	-0.52457300	-2.29666900
O	1.21559300	-2.19646200	-0.06016200
O	1.86179600	1.48349700	-0.97447300
O	-0.60338500	2.26537400	0.05803500
H	-1.43584500	2.56542200	-0.33808900
H	-0.18707300	3.01682800	0.51027800
H	-0.88915000	1.09952200	2.67131600
H	0.44522300	0.37402400	3.07573400
H	-1.13364100	-1.88552900	2.06747200
H	-1.96680600	-2.05428700	0.74641100
H	0.11287400	-1.04924500	-2.88579100
H	1.49068300	-0.29283600	-2.76723300
H	1.84034100	-2.51395500	-0.73106200
H	0.79781200	-2.96366600	0.36018200
H	2.79564500	0.60488700	1.66426800
H	2.78218400	-0.94183600	1.35754000
H	1.67661900	2.26340200	-1.52086500
H	2.82099600	1.41252700	-0.85304000
O	-1.54868900	0.01280100	-0.94725000
H	-2.35731600	0.01817100	-1.49112900

$[PaO(H_2O)_7]^{3+}(H_3O)^+$

Sum of electronic and thermal Free Energies=

-1128.433191

Pa	0.04656600	0.01542200	0.22823000
O	0.33862200	0.77294000	2.61168200
O	2.37923500	-0.12203100	1.04200600
O	-1.07512600	-1.77196600	1.51933300
O	0.30086500	-0.67515900	-2.14772500
O	1.22373100	-2.22803200	-0.10502000
O	1.59420500	1.53458100	-0.96353900
O	-0.66951800	2.35005000	0.52035600
H	-1.56609800	2.65199900	0.32093200
H	-0.14308000	3.11382400	0.79616500
H	-0.33181700	1.32654400	3.03729100
H	0.79527000	0.28188100	3.30973500
H	-0.80846400	-2.15969400	2.36554000
H	-2.02378500	-1.92483200	1.41158200

H	-0.45692900	-0.96749200	-2.67337100
H	0.95593200	-0.29960900	-2.75289000
H	1.31634900	-2.52168600	-1.02289600
H	1.01666700	-3.00806600	0.42892200
H	2.87204800	0.49139500	1.60629700
H	2.89350200	-0.93350100	0.91613400
H	1.35066200	2.27438100	-1.53846400
H	2.55926800	1.48851100	-0.91411400
O	-4.06238900	-0.07724700	-1.19146400
H	-3.06999700	-0.01768600	-0.87138500
H	-4.20316300	-0.84650500	-1.77128700
H	-4.33525800	0.73227700	-1.65827800
O	-1.69973900	0.04109200	-0.37752400

$[PaO(H_2O)_7]^{3+}$

Sum of electronic and thermal Free Energies=

-1051.612410

O	-0.17462800	-2.24786300	1.09566100
H	-0.78974400	-2.86822700	0.68253000
H	-0.35016600	-2.26271500	2.04640600
O	2.09468400	-1.79773100	-0.41489100
O	0.05971300	2.44257400	-0.01385700
O	-0.04188300	0.35662300	2.39761400
O	-0.41346900	-1.70242700	-1.66765100
O	2.34673800	1.10081900	-1.09046500
O	0.04758300	0.93951000	-2.35132800
H	2.10647600	-2.57057200	0.16759900
H	2.99383100	-1.64809300	-0.73643500
H	-0.74455600	2.88720600	0.28572800
H	0.78633800	3.07977200	0.01664400
H	-0.90217400	0.61074600	2.75780500
H	-1.32615100	-1.87105200	-1.93707700
H	3.23038500	1.26146000	-0.73256600
H	-0.34752900	1.81783100	-2.43830600
H	0.63117400	0.54068700	3.06701900
H	0.15962200	-2.36625900	-2.07650100
H	2.33771900	1.33618200	-2.02760200
H	-0.29015800	0.39346200	-3.07354200
Pa	0.31415000	-0.01520300	-0.02231000
O	-1.47320800	0.18051200	0.04935300

$[PaO(H_2O)_7]^{3+}(H_2O)$

Sum of electronic and thermal Free Energies=

-1128.062691

Pa	-0.17749300	0.10758400	0.29639200
O	0.60597800	0.52957100	2.66432900
O	2.30285000	-0.36638200	0.83671800
O	-1.36411200	-1.40473200	1.88755400
O	-0.14027900	-0.38361400	-2.17310000
O	0.50142600	-2.29970600	-0.00595900
O	1.45282600	1.51666200	-1.03734100
O	-0.49984700	2.47962100	0.94213600
H	-1.27975400	2.97798700	0.66486200
H	0.04696700	3.05319000	1.49503200
H	0.15807200	0.95497600	3.40721400
H	1.54051100	0.41255900	2.88375900
H	-1.05762600	-1.59020300	2.78603000
H	-2.32600200	-1.49609300	1.87654000
H	-0.96226800	-0.18310900	-2.63993600
H	0.20611200	-1.23752000	-2.53019500
H	0.69191000	-2.68842000	-0.89611300
H	0.00669900	-2.95147800	0.50921500
H	2.93951700	0.16521200	0.33577900
H	2.56763900	-1.29479600	0.75951400
H	1.51496800	2.47723200	-0.93946400
H	1.42780800	1.32247500	-1.98647400
O	0.81555500	-2.86489600	-2.59822400
H	0.22652200	-3.52546700	-2.98662100
H	1.68836400	-3.01693900	-2.98390300
O	-1.89414900	0.42252600	-0.14918200

$[Pa(H_2O)_9]^{5+}(H_2O)^F$

Sum of electronic and thermal Free Energies=

-1205.215393

O	-1.39214500	-0.21307900	1.88937100
H	-1.76587300	-1.00442000	2.31088900
H	-1.77748200	0.57373600	2.31015100
O	1.31443600	-0.69129200	2.16828000
O	0.71059800	0.99128400	-1.87107000
O	1.68408900	1.65114700	0.62882800
O	-0.98253800	1.75672300	0.12571900
O	-0.61128300	-2.39506300	0.69757500
O	2.50423000	-0.55373000	-0.49009900
O	0.39261500	-1.70491200	-1.75056600
H	0.91541800	-0.56711400	3.04721600
H	2.19071800	-1.09878300	2.28901100
H	0.12528300	1.65403100	-2.27544200
H	1.53504400	0.96181100	-2.38622900

H	2.00649800	1.85784600	1.52356400
H	-1.96947600	1.88936200	-0.17444300
H	-1.42385600	-2.83238800	0.38904900
H	3.25895800	0.00370700	-0.23613900
H	0.56314800	-1.46620100	-2.67800100
H	1.95569200	2.37494300	0.03932000
H	-0.64463000	2.58686400	0.50391300
H	-0.09852400	-3.04777300	1.20597800
H	2.84538900	-1.36734600	-0.89964700
H	0.40698000	-2.67556100	-1.68817000
Pa	0.21921600	-0.20310800	0.12219400
O	-3.34636100	1.66797500	-0.69384500
H	-1.91184600	-1.00131700	-1.80981300
H	-2.59933200	0.11084900	-0.93132500
H	-4.07237200	1.77191900	-0.06041400
H	-3.58561200	2.16128300	-1.49282200
O	-1.82067700	-0.46280400	-1.00562200

$[Pa(OH)(H_2O)_8]^{4+}(H_3O)^+$

Sum of electronic and thermal Free Energies=

-1205.230325

O	-1.32778800	0.19716600	1.89548700
H	-1.85308400	-0.51112600	2.29841100
H	-1.58530700	1.04193300	2.29740000
O	1.28645000	-0.80739000	2.04940700
O	0.98083600	1.02461300	-1.97509800
O	1.94525300	1.65140600	0.59250600
O	-0.62535600	2.21970400	0.14065100
O	-0.91461600	-2.10075900	0.66078600
O	2.52276200	-0.67260000	-0.45669600
O	0.48914000	-1.75859100	-1.68373800
H	0.94862700	-0.67851600	2.95099900
H	2.11108500	-1.31723700	2.09941800
H	0.47925500	1.64641900	-2.52643600
H	1.85669200	0.91223900	-2.37831200
H	2.30678100	1.79951500	1.48103200
H	-1.45765400	2.51815300	-0.25639600
H	-1.76032000	-2.41104800	0.30074200
H	3.33287600	-0.22185900	-0.17380900
H	0.67219800	-1.62320500	-2.62606500
H	2.33066200	2.31474300	-0.00070300
H	-0.18005700	2.98445400	0.53736100
H	-0.55919800	-2.79420900	1.23952600
H	2.76082100	-1.50725600	-0.89005200
H	0.19499700	-2.67465000	-1.56966000
Pa	0.21128600	-0.02504900	0.04420700
O	-4.35268800	0.28211500	-0.36587000
H	-1.74724000	-0.18262600	-1.96813600
H	-3.38435800	0.24330500	-0.55645700
H	-4.87528700	0.32276700	-1.19064300
H	-4.56731100	1.03954800	0.21278200
O	-1.51793500	0.02868100	-1.04490600

$[Pa(OH)(H_2O)_8]^{4+}$

Sum of electronic and thermal Free Energies=

-1128.419436

O	-0.23618000	-2.09474700	1.04540500
H	-0.71252800	-2.80769300	0.59626200
H	-0.35042200	-2.20494800	2.00108500
O	2.17071400	-1.63662400	-0.20666800
O	0.33255800	2.41057500	0.17246600
O	2.36793800	0.71782200	1.42207200
O	-0.12572100	0.41595700	2.29883300
O	-0.39579900	-1.67421900	-1.58340200
O	2.32120200	1.01741800	-1.18390000
O	-0.02070800	0.95361000	-2.24938400
H	2.14513200	-2.49772600	0.24037300
H	2.96527000	-1.59955400	-0.76208400
H	-0.39197000	2.90924200	0.58102400
H	0.98298200	3.04619500	-0.16379400
H	3.04866000	0.10256600	1.73396400
H	-1.02107700	0.50639300	2.65881900
H	-1.31656200	-1.79431600	-1.86173100
H	3.20004300	1.22440400	-0.83321200
H	-0.41964600	1.82731800	-2.37770100
H	2.57016000	1.59353100	1.78393400
H	0.51300000	0.53473000	3.01749400
H	0.15244600	-2.31109700	-2.06765100
H	2.29411900	1.22556200	-2.13023200
H	-0.28468300	0.39987800	-2.99870600
Pa	0.41188100	0.01879400	-0.01066500
O	-1.52628300	0.31588100	0.03380000
H	-2.48220600	0.49900700	0.00725500

$[PaO(H_2O)_8]^{3+}(H_3O)^+$

Sum of electronic and thermal Free Energies=

-1204.893046

O	-0.20113700	2.32224000	0.99884800

H	0.12028800	3.10359000	0.52871300
H	-0.11516900	2.48349500	1.94856300
O	-2.32178000	1.17576700	-0.39748900
O	0.44346200	-2.29659100	0.32105100
O	-1.99367800	-1.08546900	1.34756400
O	0.29083400	-0.10625600	2.41317200
O	0.27172200	1.88983000	-1.62059500
O	-1.67941600	-1.50085800	-1.25800900
O	0.66259900	-0.86774700	-2.20826000
H	-2.55001200	2.02734600	0.00336500
H	-3.04380800	0.90654200	-0.98309900
H	1.24779600	-2.62421200	0.74743200
H	-0.04269400	-3.05493000	-0.03241300
H	-2.84369000	-0.68137500	1.57209600
H	1.16107500	0.01273100	2.81747900
H	1.13645300	2.24580200	-1.86951900
H	-2.52888600	-1.87441700	-0.98752000
H	1.24667600	-1.63862200	-2.23166100
H	-1.95626500	-1.95311000	1.77226000
H	-0.34776700	-0.31830700	3.10763500
H	-0.39389000	2.29192700	-2.19722500
H	-1.53369400	-1.68108100	-2.19746800
H	0.94114100	-0.27883700	-2.92247100
Pa	-0.14363400	0.05264200	0.00873300
O	4.12187200	-0.23154300	0.15838400
H	4.58513000	0.24801900	-0.54935800
H	4.55145600	-0.03847800	1.00914700
H	3.07959000	-0.00016700	0.17882000
O	1.70014300	0.20483400	0.18165400

$[PaO(H_2O)_8]^{3+}$

Sum of electronic and thermal Free Energies=

-1128.058039

O	2.51601900	-0.51974600	0.01878300
H	3.18331700	0.15929800	-0.14627700
H	2.83155800	-1.34276800	-0.37771800
O	0.92740300	-0.05620900	2.23120100
O	-2.14764600	-0.55429200	-1.06254500
O	-0.88996300	-2.00075300	1.19949000
O	0.56001900	-2.17894000	-1.11024400
O	1.47194200	2.05243400	0.12227100
O	-1.77475500	0.46999200	1.57255000
O	-1.34379800	2.17433800	-0.37961100
H	1.85261000	-0.26477900	2.42251800
H	0.51241600	0.29222400	3.03176500
H	-2.30682600	-0.72131900	-2.00140300
H	-2.99704400	-0.58603000	-0.60232200
H	-0.46419000	-2.33942500	1.99861600
H	0.74733900	-2.29473900	-2.05106900
H	1.82001100	2.55300000	-0.62805500
H	-2.16378500	-0.14370900	2.20870700
H	-2.00667200	2.19354400	-1.08302800
H	-1.59797100	-2.61469900	0.96338800
H	0.39597500	-3.04725800	-0.71996800
H	1.71947100	2.51794100	0.93332000
H	-2.22681100	1.32316800	1.62348300
H	-0.87855800	3.02083900	-0.41380000
Pa	0.04388800	0.03311200	-0.12127000
O	0.37894900	0.39836500	-1.85722200

$[PaO(H_2O)_8]^{3+}(H_2O)$

Sum of electronic and thermal Free Energies=

-1204.514774

O	-0.44099100	2.37785700	0.99978500
H	-0.13616400	3.16277500	0.52486000
H	-0.15556900	2.46273900	1.91941500
O	-2.41894900	1.10228200	-0.31858000
O	0.66471600	-2.19386000	0.19159300
O	-1.85968500	-1.23886200	1.35552700
O	0.36213600	0.10647700	2.35290200
O	-0.00012500	2.02017300	-1.71639600
O	-1.78629600	-1.40809800	-1.32245800
O	0.58678500	-0.78500500	-2.35977700
H	-2.66273300	1.93060600	0.11757500
H	-3.14676600	0.81158200	-0.88441400
H	1.49106900	-2.43406300	-0.29676000
H	0.10059800	-2.97882000	0.18083800
H	-2.71941300	-0.85164800	1.57127200
H	1.27339800	0.15124700	2.67095300
H	0.82915400	2.40722400	-2.02715500
H	-2.43995600	-1.98738200	-0.91043500
H	1.36226100	-1.39281800	-2.33539700
H	-1.73084500	-1.98549200	1.95559000
H	-0.19461700	-0.20698000	3.07698000
H	-0.71624900	2.37372900	-2.26115100
H	-1.53328500	-1.77083400	-2.18214100
H	0.72147000	-0.16803200	-3.09056200
Pa	-0.13184100	0.09688000	-0.10260100

O	2.53398000	-2.55294300	-1.68373400
H	3.45030800	-2.25863100	-1.59985100
H	2.56640000	-3.42067300	-2.10789800
O	1.63911000	0.44609600	-0.02557600

$[PaO(H_2O)_6]^{3+}(H_2O)'$

Sum of electronic and thermal Free Energies=

-1051.604756

O	-2.47175200	-0.51831900	-0.20758000
H	-2.74331500	-1.35066400	-0.61569300
H	-3.10544700	0.16479200	-0.46364000
O	-0.03681300	-0.14756600	2.80749900
O	1.76078700	1.72635600	-0.30704600
O	-1.35001300	2.08091100	-0.29146500
O	-0.38549600	-2.30066700	-0.65929800
O	2.15505800	-0.96290200	-0.42511600
H	-0.83363800	-0.48648300	3.23834700
H	0.71445400	-0.60153800	3.21373000
H	1.78050000	2.21604400	-1.14022400
H	2.13226700	2.29963000	0.37819400
H	-1.39532300	2.48211700	-1.16938400
H	-0.37391000	-2.55563700	-1.59170500
H	2.87342800	-0.38025300	-0.70527400
H	-1.61472900	2.74470900	0.36088800
H	-0.38778100	-3.10650000	-0.12358400
H	2.29788800	-1.83691500	-0.81011800
Pa	-0.05348000	0.02998400	0.13030000
O	-0.09573800	0.21365200	-1.65731000
O	0.77090000	0.02305800	-4.49431800
H	1.24426900	0.85672500	-4.58524300
H	0.25826600	0.14282900	-3.68486400
